# The Viral Origin of Human Breast Cancer: From the Mouse Mammary Tumor Virus (MMTV) to the Human Betaretrovirus (HBRV)

**DOI:** 10.3390/v14081704

**Published:** 2022-08-01

**Authors:** Generoso Bevilacqua

**Affiliations:** Department of Translational Research, University of Pisa, 56126 Pisa, Italy; generoso.bevilacqua@outlook.it

**Keywords:** HBRV, MMTV, HMTV, human Betaretrovirus, mouse mammary tumor virus, human mammary tumor virus, breast cancer, PBC, primary biliary cholangitis, etiology

## Abstract

A Human Betaretrovirus (HBRV) has been identified in humans, dating as far back as about 4500 years ago, with a high probability of it being acquired by our species around 10,000 years ago, following a species jump from mice to humans. HBRV is the human homolog of the MMTV (mouse mammary tumor virus), which is the etiological agent of murine mammary tumors. The hypothesis of a HMTV (human mammary tumor virus) was proposed about 50 years ago, and has acquired a solid scientific basis during the last 30 years, with the demonstration of a robust link with breast cancer and with PBC, primary biliary cholangitis. This article summarizes most of what is known about MMTV/HMTV/HBRV since the discovery of MMTV at the beginning of last century, to make evident both the quantity and the quality of the research supporting the existence of HBRV and its pathogenic role. Here, it is sufficient to mention that scientific evidence includes that viral sequences have been identified in breast-cancer samples in a worldwide distribution, that the complete proviral genome has been cloned from breast cancer and patients with PBC, and that saliva contains HBRV, as a possible route of inter-human infection. Controversies that have arisen concerning results obtained from human tissues, many of them outdated by new scientific evidence, are critically discussed and confuted.


**Index**

*Preamble*

*Preface*
Part I1. Introduction 1.1. *Cancer and Its Many Names*1.2. *Breast and Mammary Gland*1.3. *Basic Concepts of Breast Oncogenesis*1.3.1. The Mammary Gland Is a Dynamic Organ, with Consequence on Carcinogenesis1.3.2. The Terminal Duct Lobular Unit Is the Site of Origin of Breast Carcinoma1.3.3. Initiation, Promotion, Progression1.3.4. Preinvasive Lesions1.3.5. Risk Factors1.4. *Sporadic and Hereditary Breast Carcinoma*1.4.1. The Knudson’s Model1.4.2. Haploinsufficiency1.5. *Human Breast Cancer Etiology Is Unknown, So Far*Part II: Mouse Mammary Tumor Virus—MMTV2. The Experimental Model of Breast Cancer: The Mouse Mammary Tumors and the Mouse Mammary Tumor Virus (MMTV), with Its Long List of Names3. The Mouse Mammary Tumor Virus3.1. *Classification*3.2. *Viral Structure and Molecular Trafficking*3.2.1. Viral Packaging3.2.2. Viral Budding3.3. *Transmission by Nursing*3.4. *Intestinal Absorption*3.5. *Cell Infection*3.6. *Molecular Structure*3.7. *Oncogenesis*3.7.1. Insertional Mutagenesis, Int, Cis3.7.2. Oncogene3.7.3. Transcription3.8. *Mouse Strains and Their Tumors*3.9. *How Many Types of MMTV?*3.10. *MMTV Can Infect Adult Mice by an Alternative Way of Entry: The Nasal-Associated Lymphoid Tissue*3.11. *Not Only Mammary Tumors*3.12. *Endogenous MMTV*3.12.1. Transposons, Retrotransposons, *ERVs*Part III: Human Mammary Tumor Virus—HMTV4. The Human Mammary Tumor Virus4.1. *HMTV in Human Milk*4.2. *HMTV in Human Cancer*4.2.1. Molecular Biology and Immunohistochemistry4.2.2. The gp52 Glycoprotein5. The ENV Sequence, the HMTV, and the Breast Carcinoma: The New York Laboratory5.1. *Env Sequences in Human Breast Carcinoma*5.2. *Env Sequence Expression*5.3. *MMTV Provirus*5.4. *MMTV-Like LTR and LTR-Env Gene Sequences*5.5. *The Superantigen (sag)*5.6. *The Interferon Signature*5.7. *MMTV Particles in Human Cells*5.8. *MMTV Proteins in Human Cells*5.9. *The HMTV—Human Mammary Tumor Virus*6. MMTV/HMTV Is Significantly Present All over the World7. HMTV Is Able to Infect Human Cells: The Vienna Laboratory7.1. *TfR1—The Transferrin Receptor 1*7.2. *HMTV Can Infect Human Cells*7.3. *HMTV Does Not Use TfR1 to Infect Human Cells*8. HMTV Is Involved in Almost All Sporadic Breast Carcinomas: The Pisa Laboratory8.1. *Stringent Laboratory Procedures Guarantee the Quality of the Result*8.2. *HMTV Is Present in the Vast Majority of Sporadic Breast Carcinomas*8.3. *Hereditary Breast Carcinomas Do Not Need a Viral Etiology*8.4. *HMTV in Saliva and the Paleoanthropological Study*9. The P14, a MMTV Signal Peptide—A Possible Tool for Vaccination Strategies: The Laboratory of Jerusalem10. The Point of View of an Epidemiologist: The Laboratory of Sidney11. HMTV in Human Neoplasia Other Than Breast Carcinoma11.1. *Non-Hodgkin Lymphoma (NHL)*11.2. *Tumors Influenced by Hormones*11.3. *Lung Cancer*12. HMTV and the Human Primary Biliary Cholangitis: The Laboratory of Edmonton13. HERVs—Human Endogenous Retroviruses14. The Leitmotiv of Contamination: No Evidence14.1. *Contamination*14.2. *HERVs*14.3. *Other Issues*15. Breast Cancer Incidence in Immunosuppressed Patients16. The Pisa Meetings16.1. *2012 (2–4 April): HMTV—Viruses and Breast Cancer*16.2. *2016 (22–23 September): HBRV—MMTV and Human Diseases*Part IV: Human Betaretrovirus—HBRV17. The Human Species Has Its First Human Betaretrovirus 18. Cross-Species Transmission19. HBRV as the Product of a Cross-Species Transmission20. The Possibility of a Contemporary Zoonosis21. Inter-Human Transmission of HBRV21.1. *Milk (Breast Feeding) Cannot Be the Answer*21.2. *Retroviruses (MMTV Included) Can Be Transmitted by Saliva in Animals*21.3. *MMTV Can Infect Adult Mice through the Nose*21.4. *HBRV Is Present in Human Saliva*22. HBRV Transmission by Saliva22.1. *Saliva Contains a Little Universe*22.2. *Saliva Spreading*22.3. *Saliva Transmits Even Prions*22.4. *What Is in Saliva That Can Diffuse HBRV*23. Breast Cancer in the Same Family: A Transmissible Agent?24. The Abundance of BC Histotypes and Biological Types Can Find a Reason in a Viral Etiology25. Oncogenesis of HMTV25.1. *Proteins with the Characteristics of Oncogene*25.1.1. The Env Protein25.1.2. The Sag, Superantigen25.1.3. The Naf, Negative Acting Factor25.1.4. The REM, Regulator of Expression/Export of MMTV mRNA25.2. *Cell Fusion*25.3. *Activation of a Second Oncogenic Virus*25.4. *HERVs*26. How Many HBRV?Part V27. Keynotes28. For the Future
*To Francesco Squartini, 1927–1992,*

*pioneer of the mouse mammary tumor biology*


*Preamble*

These notes synthesize most of what is known about the possible viral origin of human breast cancer and would, perhaps, be useful also to scientists who are not strictly involved in the field. For this reason, the paper recalls some basic concepts, of breast cancer biology and cancerology in particular.

The whole of almost one century of study demonstrates the existence of HBRV, the human homologue of MMTV, endemic in the human species, with high probability as the etiologic agent of breast cancer (BC) and primary biliary cholangitis (PBC).

*Preface*

This is the story of a virus grown in the mouse and passed to humans thousands of years ago, which has many names, causes mammary tumors in mice, and is strictly related to BC and to PBC.

The story begins in the countryside of Maine almost a century ago, in a golden age for science: inbred mice are raised to study the genetic transmission of diseases, viruses are discovered, Oswald Theodore Avery, Colin Munro MacLeod, and Maclyn McCarty individuate DNA as the transforming principle able to modify the characteristics of bacteria (Pneumococcus type III) [1].

In 1908 Ellerman and Bang [2] discovered the virus of chicken leucosis, the first tumor inducing virus, followed by the Rous sarcoma virus in 1911 [3]. In 1938 Siemens made the first electron microscope commercially available. This revolutionary technology finally allowed researchers to see the viruses, to classify them on the basis of their structure, and to describe the stages of their development. In 1945 Porter, Claude and Fullam obtained the first picture of cells derived from chick-embryo tissue [4]. Wilhelm Bernhard was a pioneer in the development of electron microscopy and in the study of the ultrastructure of viruses [5,6].

**Part I.**

## 1. Introduction

### 1.1. Cancer and Its Many Names

The number of cells of an adult human organism has to remain constant; in the single tissue so many cells die, while so many cells are born. This balance is guaranteed by sophisticated biomolecular processes involved in controlling cell-cycle progression, apoptosis, DNA repair, degradation of proteins, assemblage of the mitotic spindle, etc. Some genes and their coded proteins (a) exert an inhibitory control on proliferation, whereas others (b) activate the process. The disarrangement of this machinery can lead to an uncontrolled cell proliferation, that is, a tumor. This result can be consequence of a damage in specific genes, with the consequent origin of mutated proteins. When the mutated gene/protein belongs to the above mentioned “a” group, they are called suppressor (or oncosuppressor) genes/proteins, whereas when they belong to the “b” group, they are named oncogenes/oncoproteins.

The Latin word *tumor* means swelling. *Neoplasia* derives from the ancient Greek words *νέοσ* (neos, new) and *πλάσσω* (plasso, to form) and means “new formation”. Cancer is the Latin word for crab. Carcinoma derives from the ancient Greek *καρκίνος* (karkìnos, again for crab). Cancer, karkinos, and crab are all linked by the Indo-European root *kar-* that means “hard”. Actually, we might say “hardoma”(!). The strict link between hardness and disease probably derives from breast cancer, one of the most frequent and distinctly visible growths, because it occurs in a superficial organ, and often very hard as a consequence of its abundant stromal component. In fact, one of the common histotypes of breast cancer was called “scirrhous”, (*σκιρός,* skiròs), with the same root.

For no evident reason, cancer indicates any malignant tumor, whereas carcinoma indicates cancers deriving from epithelial cells. The two words are often wrongly used as synonyms. Sarcoma derives from *σάρξ* (sarcs, flesh) and indicates cancer of mesenchymal tissues.

### 1.2. Breast and Mammary Gland

Breast is made by a more or less abundant quantity of adipose tissue hosting the mammary gland tree. From the nipple a dozen of galactoforous (lactiferous) ducts penetrates the subcutaneous fat giving origin, by a series of dichotomous divisions, to segmental and subsegmental ducts. Each of them ends with a short and thin peduncle that holds a bunch (lobule) of very small glandular acini: the terminal duct lobular unit (TDLU). Milk is produced in the lobule and then transported to the nipple by the duct system.

### 1.3. Basic Concepts of Breast Oncogenesis

#### 1.3.1. The Mammary Gland Is a Dynamic Organ, with Consequence for Carcinogenesis

The mammary gland is characterized by an amazing activity. During a woman’s reproductive life, each month there is an intense proliferation of the TDLUs, which prepare themselves to produce large quantities of milk. In contrast, after the menstruation, lobules disappear following an apoptotic program. The fact that mammary epithelial cells are *labile*, i.e., divide all the time, makes them highly susceptible to carcinogenetic agents, which more easily can reach the cell DNA during the mitotic division. *Stable* (quiescent) cells spend most of their life in G_0_, and then have a low incidence of cancer (hepatocytes). *Permanent* cells are considered terminally differentiated and, therefore, are non-proliferative; for instance, neurons only exceptionally participate in the formation of tumors of the nervous system. Labile cells are more prone to develop cancer, also because of their high proliferative rate facilitates the occurrence and accumulation of the gene mutations necessary for the neoplastic transformation and progression.

#### 1.3.2. The Terminal Duct Lobular Unit Is the Site of Origin of Breast Carcinoma

TDLU is the dynamic part of the glandular tree, while ducts are stable. As expected, TDLU is the site of origin of a carcinoma, both in a woman and in the mouse.

#### 1.3.3. Initiation, Promotion, Progression

Human and murine mammary glands are similar, aside from their number (ten in the mouse). In fact, much of what we know on the pathogenesis of BC derives from the murine model. The natural history of a carcinoma has three main steps: (a) *initiation,* the moment of the transformation of normal cells in neoplastic cells by a carcinogenetic agent/process; (b) *promotion,* the stimulus to proliferation exerted not only on the transformed cells but on the entire cell compartment to which they belong; (c) *progression,* the whole of all the phases that characterize the entire life of the process. The concept of progression was created by Leslie Foulds in the ‘50s, mainly by the observation of mouse mammary neoplasia [7]. Estrogens are powerful promoting agents, i.e., able to stimulate cell proliferation. Progression recognizes different temporal phases characterized by different bio-molecular mechanisms: *early*, with the occurrence of preinvasive lesions; *intermediate*, that is the invasion of the surrounding normal tissues; *late*, with the metastasis. The whole of all the processes at the base of promotion and progression represents the *pathogenesis* of the disease, in short, how the disease develops.

#### 1.3.4. Preinvasive Lesions

Before the appearance of a clear invasive carcinoma, promotion and progression give origin to typical and atypical *preinvasive* lesions, secondary to an excessive/abnormal estrogen stimulation: UDH (usual ductal hyperplasia), ADH (atypical ductal hyperplasia), DCIS (ductal carcinoma in situ). ADH and DCIS are considered obligatory precursors of the invasive carcinoma [8].

#### 1.3.5. Risk Factors

As expected from the abovementioned, BC risk factors are mainly related to estrogen stimulation and to the number of proliferative cycles. An early menarche, a late menopause, and nulliparity are risk factors because they increase the number of menstrual cycles and, as a consequence, the exposition to estrogens and the number of cell cycles. Obesity is a risk factor because of the endogenous production of estrogens in the adipose tissue deposits.

### 1.4. Sporadic and Hereditary Breast Carcinoma

The vast majority of BC is “sporadic”, because it occurs randomly, and is not linked to any identified etiological factor. Conversely, according to the American Cancer Society 5–10% of BCs are induced by inherited highly penetrant pathogenic mutations; therefore, they are named “hereditary” (HBC). The mutations affect a group of tumor suppressor genes (TSG) and are transmitted in an autosomal dominant way from one parent. Two TSGs, BRCA1 and BRCA2 (BReast CAncer), are responsible for 80–90% of cases of “single gene” hereditary breast carcinoma. Women with a mutation in the BRCA1 face as high as 80% lifetime risk of developing breast cancer.

#### 1.4.1. The Knudson’s Model

According to Knudson’s model [9], both alleles of a TSG conferring susceptibility to breast cancer (as BRCA1) need to be mutated to initiate carcinogenesis. One allele is inherited already mutated (first hit), whereas the second one is mutated during the lifetime (second hit). However, there are cases in which the second mutation cannot be demonstrated. Recently, to explain this discrepancy, a great deal of attention has been given to the status of the protein coded by the TSG, applying to hereditary tumors the concept of haploinsufficiency, a known causative mechanism of non-neoplastic diseases [10].

#### 1.4.2. Haploinsufficiency

Haploinsufficiency occurs when one allele of a gene is inactivated but the protein produced by the remaining functional allele is not able to assure the physiological functions. One possibility is that the mutated allele is dominant negative so that the mutant protein interferes with the wild-type protein. The result is similar to the loss of heterozygosity (LOH) caused by the Knudson’s hypothesis, without the inactivation of the second allele. Several studies support the hypothesis that the BRCA1 two-hit mechanism is not the only one, and haploinsufficiency is involved [11]. Moreover, BRCA1 haploinsufficiency is unique to normal human-breast epithelium, explaining why neoplastic transformation in hereditary breast tumors is limited to the mammary gland in most cases [12,13].

In summary, the hereditary transmission of mutated TSG can be considered an etiological factor for HBC.

### 1.5. Human Breast Cancer Etiology Is Unknown, So Far

While the pathogenesis of the disease is known in detail, no etiological agent has ever been identified for sporadic BC, to date.

Unless further specified, breast cancer and BC refer only to sporadic tumors throughout the article.

**Part II: Mouse Mammary Tumor Virus—MMTV**

## 2. The Experimental Model of Breast Cancer: The Mouse Mammary Tumors and the Mouse Mammary Tumor Virus (MMTV), with Its Long List of Names

The murine model of mammary tumors induced by the Mouse Mammary Tumor virus has a central role in cancer biology, not only in that related to the breast. The stage of the first chapter of its fascinating story was the young Roscoe B. Jackson Memorial Laboratory in Bar Harbor, Maine, under the direction of Clarence Cook Little. Jackson, an engineer, was general manager and treasurer of the Hudson automaker and an enthusiastic supporter of cancer research. Funded by Jackson and others, Little’s new laboratory was aimed to study cancer genetics in mice. This happened in 1929, the same year of Jackson’s early death, a victim of influenza during a trip in France, according to the New York Times (21 March 1929, page 31). The laboratory was then named after him and now is known also as “Jax” [14]. The novelty and the strength of the new institution was its project to study the genetics of tumors using inbred strains of mouse, a mammal with a very high reproductive rate.

The existence of *spontaneous* mammary tumors in mouse has been known since the beginning of the 20th century, described by Hugo Apolant [15], and Magnus Haaland [16]. Moreover, in 1927 Haaland hypothesizes the infectious etiology of mouse tumors and states “the infection theory of cancer seemed to be more of a reality than even before” [17].

In 1933, Jax announced “the existence of non-chromosomal influence in the incidence of mammary tumors in mice” [18], suggesting that an environmental factor could have a role in causing these neoplasms. In 1935, Murray and Little published that the extra-chromosomal influence was about six times that of the possible chromosomal influence [19].

The following year, John Joseph Bittner entered the scene as protagonist. In his first paper [20], he gave a name to the “non-chromosomal influence”: *nursing*. High-tumor strains may be changed to low-tumor strains by foster-nursing by females with a low tumor incidence [21,22]. Bittner also demonstrates that the “influence” had no effect on the development of primary lung cancer and began to give weight to the concept of influence: it could be a hormone, a chemical or a virus. The hypothesis of a virus made its appearance in 1939 [23].

In 1940, Bittner stated that a maternal “influence” can be transmitted by way of the mother’s milk: the *milk influence* [24].

In 1942, “nursing” became “milk-influence”, and the word “*agent*” was introduced: “probably a colloid of high molecular weight” [25] and “it is possible that the active agent is a colloid of high molecular weight” [26]. Woolley, Law, and Little introduced the acronym MTI—mammary tumor *inciter* [27]; the inciter was present in normal spleen, thymus, and mammary tissue of high cancer incidence animals [28], and also in blood [27]; moreover, the inoculation of whole blood could increase the incidence of mammary tumors [29].

In 1949, Graff, Moore, Stanley, Randall, and Haagensen isolated from milk the “*mouse mammary carcinoma virus*” and saw the viral particles at the electron microscope using the technique of the shadow casting with gold [30]. In the same year, Mann spoke of “Bittner virus” [31] and next year Barnum and Huseby introduced the *milk agent virus* [32].

Finally, in 1958, we have the birth of the *mouse mammary tumor virus*, as named by Phyllis Blair [33,34]; in 1965, the same Blair used the acronym *MTV*, mammary tumor virus [35].

After a long gestation, more than 30 years, MMTV, mouse mammary tumor virus, became very popular in the 1970s.

## 3. The Mouse Mammary Tumor Virus

### 3.1. Classification

The mouse mammary tumor virus (MMTV) is a Betaretrovirus. A Retrovirus is a virus able to insert a copy of its genomic RNA into the DNA of the infected cell. This result needs the intervention of the enzyme “reverse transcriptase”, which is able to obtain a DNA copy of the viral RNA.

MMTV is one of the five virus species that constitute the genus *Betaretrovirus*, present in vertebrates, with the genome composition ssRNA-RT. The other four are the Jaagsiekte sheep retrovirus (JSRV), the Langur virus (LNGV), the Mason–Pfizer monkey virus (MPMV), and the Squirrel monkey retrovirus (SMRV). MMTV is the etiological agent of the mouse mammary tumors. JSRV causes the *jaagsiekte* or ovine pulmonary adenocarcinoma or ovine pulmonary adenomatosis; *jaagsiekte* is an Afrikaans word composed of *jaag* (chase) and *siekte* (sickness) to indicate the breathing difficulties of the infected animals when chased, caused by the severe alterations of the lung parenchima. LNGV, or Po-1-Lu, is an endogenous virus of Langurs, a species of primate in the family of Cercopithecidae, subfamily Colombinae. The name PO-1-Lu derives from its species of isolation (Presbytis obscurus—PO) and from the fact that it was originally isolated by co-cultivating cells from langur and bat lung (Lu). MPMV causes immune deficiency in Asian macaques. SMRV was isolated from a New World squirrel monkey.

### 3.2. Viral Structure and Molecular Trafficking

Our understanding of virus assembly is partial and derives from studies on several retroviruses, such as the *Betaretroviruses* MMTV and MPMV and the *Lentivirus* HIV, and from research about the physiological mechanisms of cells.

Before going into some details, the prominent role of the viral Gag polyprotein (group antigen) has to be mentioned. Gag assembles at the plasma membrane from where both viral packaging and budding directs. Sometimes the virus is produced in cytoplasmic vacuoles/vesicles.

#### 3.2.1. Viral Packaging

- Bernhard described two types of MMTV particles, A and B [5,6]. The B particle is THE virus, able to infect cells. The A particle is its intracytoplasmic precursor, the “immature” form. A particles reside in the cytoplasm, usually grouped in a dense matrix in the Golgi area. They are round, measure approximately 70 µm and consist of a concentric double membrane with an electron lucent center, similar to “doughnuts” Bernhard says. The mature virion, the B particle, is found out of the cell, in mammary gland lumina and in intercellular spaces, but also inside the cell, in large intracytoplasmic vacuoles, which then release their content outside. B particles measure around 105 µm and have an eccentric electron dense nucleoid. Moreover, they are crowned with prominent “spikes”, surface glycoproteins, which can be easily highlighted at the ultrastructural level by negative staining.
- Viral particles are formed and then excreted into the mammary gland lumen or into intercellular spaces. Assemblage and excretion go hand in hand and are achieved through an amazing activity of the host-cell organelles and molecules, with viral proteins playing a leading role. The process of particle manufacturing requires high specificity to ensure the continuity of the viral life cycle. A crucial point, in fact, is the need for only the viral genomic RNA (gRNA) to enter into the particle; “unfortunately” viral gRNA represents only ~1% of the total cellular RNA. This amazing “fishing” must select it in the “crowd” of non-viral and non-genomic RNA molecules present in the cytoplasm and almost simultaneously must pack it into the viral particle, the virion. The process is orchestrated by the viral polyprotein Gag.
- Retroviruses contain two copies of unspliced full-length gRNA. This means that for each virion, two copies of gRNA need to be associated into a dimer by means of non-covalent links. Then, the dimer is selectively packaged into the forming viral particle. Two factors drive the process: specific gRNA sequences and Gag polyprotein, able to identify these sequences and to bind them [36,37,38,39].

#### 3.2.2. Viral Budding

- The events summarized above give origin to the A particles. Later, they become type B particles, acquiring an external membrane, provided by cell membranes through a process of “budding” on the cell surface or in the lumen of cytoplasmic vacuoles and vesicles. Budding is a critical moment, which takes place due to sophisticated molecular events, quite usual in different aspects of the cell life and utilized by almost all types of cellular membranes.
- ESCRT, the endosomal sorting complex required for transport, plays a main role in membrane remodeling, from the setting up of exosomes, to membrane repair, to virus budding. The main members of this protein pathway are ESCRT-0, -I, -II, -III, ALIX, and the AAA-type ATPase VPS4 (vacuolar protein sorting). Gag polyprotein with the collaboration of ALIX (PDCD6IP, interacting partner) recruits ESCRT-I, a heterotetramer able to interact with ESCRT-II to favor the formation of the membrane bud. ESCRT-II activates ESCRT-III. Some of the numerous ESCRT-III proteins constrict the bud neck and bring it close to the fission [40,41,42,43,44].
- Modern technology allowed the understanding, still partial, of the mechanism necessary for the dynamic activity of the cell membranes, giving a meaning to electron microscopical images that 40 years ago were considered only “unusual findings of viral production” [45].

### 3.3. Transmission by Nursing

#### Infection Requires a Long Period of Breastfeeding

In mice, virus is produced mainly in the acini of the mammary gland; hence, the milk of strains with a high incidence of mammary tumors has plenty of particles. They are present as classic round B particles or with a “head and tail” morphology, well evident at the electron microscope when the negative staining is used [46]. However, a large part of the virus is destroyed by the acid environment. The particles swell taking the shape of a “signet ring” with the nucleoid compressed at the periphery, the capsid breaks, the virus looks like a “comet” with a well-defined head and a large burst tail. Numerous nude nucleoids are present, often in small groups. These morphological findings [47], so simple if compared with the molecular events just mentioned, contribute to explain why the infection of the newborn mouse requires a long period of breastfeeding. In fact, at any lactation the largest part of the virus is destroyed in the stomach so that the critical threshold for the infection, in terms of quantity of particles, is reached only after a long nursing.

### 3.4. Intestinal Absorption

An ultrastructural paper of 1989 [47] shows some details of the fate of MMTV in the ileum of the newborn mouse. A large quantity of viral particles reaches the ileum, most of which badly damaged by the gastric juice [48]. At the level of Peyer’s patches, the virus binds the microvilli of enterocytes and enters their cytoplasm by endocytosis. The involved cells are newborn-type and adult type epithelial cells, and M cells. These last ones were described in 1974 in the Peyer’s patches and were identified also in isolated lymphoid follicles, in colonic patches, and in nasopharyngeal associated lymphoid tissue; M is for “microfold” [49]. M cells have a transcytosis function and transfer luminal particles and antigens to dendritic cells (DCs) [50].

In the Peyer’s patches the infected DCs are activated by MMTV also by mean of the interaction of the viral Env with its toll-like receptor (TLR4). At the same time, there is an increased expression of the receptor TfR1/CD71 (transferrin receptor protein—cluster of differentiation), important for the entrance of the virus, thereby potentiating the infection. The spreading of the infection requires an intense proliferation of lymphocytes, mainly B cells, and this goal is assured by the viral Sag (superantigen). DCs produce cytokines that stimulate B cell proliferation. DCs and B cells, as antigen-presenting cells, express on their surface MHC class II proteins (major histocompatibility complex), which present Sag to T cells inducing their proliferation. In turn, T cells secrete cytokines that recruit and stimulate more DCs, B cells and T cells. Similar events take place also in the local lymph nodes where DCs migrate. In such a way, the lymphocyte compartment is well expanded and can start the infection of the entire animal [51,52,53,54]. It was reported that B cells migrate preferentially to the mammary gland and also to lung and liver, whereas T cells go to lymph nodes, spleen and intestine [55].

The intense activity of a perfectly working immune system is essential for the expansion of the viral population and for the infection of the host. In contrast, once ingested by newborn mice, MMTV stimulates a status of tolerance towards its antigens. For this goal, the intestinal microbiota plays a major role, through the bacterial lipopolysaccharide (LPS). MMTV-bound LPS stimulates the Toll-like receptor 4 (TLR4) with the subsequent activation of the interleukin 6 (IL-6), which induces the production of cytokine IL-10, which has an immunosuppressive effect [56,57].

### 3.5. Cell Infection

How lymphocytes transmit MMTV to the epithelium of the mammary gland is not known. From the process of intestinal absorption and from cell cultures it can be inferred that MMTV enters mammary epithelial cell after that its Env protein binds to a cell membrane receptor, possibly TfR1 (transferrin receptor), a protein involved in iron metabolism [58]. Generally, the viral particle gets inside the cytoplasm by means of a process of endocytosis.

### 3.6. Molecular Structure

In 1996, Robin A. Weiss classified retroviruses into “simple”—owning only *gag*, *pol* and *env* genes—and “complex”, when containing also non-structural genes with regulatory or auxiliary functions [59].

To export incompletely spliced mRNAs from the nucleus, simple viruses (as MPMV) utilize a cis-acting RNA element (CTE, constitutive transport element), which directly interacts with specific proteins, whereas complex viruses utilize trans-acting proteins encoded by themselves, which drive nucleo-cytoplasmic transport of incompletely spliced viral transcripts, such as Rem.

MMTV, once considered “simple”, has the characteristics of being “complex”. In fact, in addition to the structural genes *gag* and *env* (necessary for its formation) and to the genes for its replication, *pro* and *pol*, MMTV has *sag* (superantigen), and the dUTPase (deoxyuridine 5′-triphosphate nucleotidohydrolase), involved in nucleotide metabolism and possibly in viral replication in non-dividing cells. Moreover, MMTV encodes a Rem (regulator of expression/export of MMTV mRNA), which is a Rev-like protein (regulator of expression of virion proteins) present in the nucleus and is involved in the export of unspliced or incompletely spliced mRNA. Finally, *Naf* (negative acting factor), a negative regulator of transcription, can be noticed. *Sag* and *Naf* share parts of their coding sequence [60,61,62,63,64,65,66].

Going into more details, the U3 region of the MMTV LTR contains an ORF encoding Sag as well as Naf. In B cells Sag expression is directed by the U3 promoter P2, even in the absence of the P1 promoter. P2 was suggested as being the major promoter used for expression of Sag in B lymphocytes, with consequent amplification of B and T cells [67,68].

### 3.7. Oncogenesis

#### 3.7.1. Insertional Mutagenesis, Int, Cis

Oncogenic viruses can induce tumors in animals in a different lapse of time, short or long: 2–3 weeks vs. months. This diversity is a consequence of a different mechanism of transformation. In the first case, the virus introduces an oncogene in the host, which is able to trigger directly the carcinogenesis. In contrast, the provirus inserts into the host genome at random without a preferred site, deregulating genes, which in their turn can initiate the neoplastic transformation. This last mechanism (insertional mutagenesis) requires more time. Retroviruses of the first group are defined as “acute” (or acute-transforming), while those of the second group are defined as “slow” (or slow-transforming or non-acute transforming) [69].

MMTV is a slow-transforming retrovirus. The genes affected by its insertion belong to the category of *Int* loci, locus of integration. The number of *Int* is quite high, and they are different in the different mouse strains. However, the first ones to be identified regard important molecular pathways: (a) *Int1* was later identified as the *Wnt1* gene (*Wingless/Int1*, Wnt/Rspo/Fgf signaling pathways), involved in embryonic process, as cell proliferation and migration; (b) *Int2* was shown to be the *Fgf3* gene (fibroblast growth factor), negative regulator of bone growth; (c) *Int3* was identified as *Notch4* (neurogenic notch homolog 4), which regulates cell-cell interaction, and; (d) *Int6*, *eIF3e* (eukaryotic translation initiation factor 3) [70,71]. Other CIS with an oncogenic activity have been identified [72].

When clusters of integrations occur in small genomic areas, these areas are named CIS (common integration site). How a CIS has to be defined is open to discussion. According to Robert Callahan et al. [71,73] “CIS means that the gene was altered by a proviral insertion in at least two independent lesions arising in different hosts”. Andrew Mason suggests “three or more independent HBRV proviral insertions arising in separate individuals (or experiments in vitro) found to be within an 88 kb range” [74]. However, CIS is defined “core” (or high frequency) when the number of insertions is at least five, while a low frequency CIS indicates a number of four or less.

Again, Holger Weishaupt et al. determine CIS using two different approaches [75]:

(a) “The first approach is based on previous Monte Carlo observations of the distribution of insertion sites in the genome, and identifies CISs as clusters of four or more integration sites within a 100 kb window, three integration sites within a 50 kb window, two integration sites within a 30 kb window.
(b) “The second approach assumes that integration sites exert the greatest effect on the transcription of genes within a 50 kb range and therefore establish CISs as network of integration sites that are linked to each other if they are within 50 kb of each other”.

Basically, to be defined as “common” the integrations have to occur with a “certain” frequency in a limited area. Callahan suggests that CIS having less than five integrations could simply occur by chance [71].

#### 3.7.2. Oncogene

Insertional mutagenesis is not the only strategy used by MMTV. Its *env* gene, in fact, can act as an oncogene. The env protein (the spike protein) plays an important role in carcinogenesis. Env is a membrane fusion protein critical for binding cell membrane receptors and penetrating into the cell, made by two parts, SU (surface unit) and TM (transmembrane protein). SU, which can be also transmembrane, hosts a ITAM sequence (immunoreceptor tyrosine-based activation motif) that has an important role in signal transduction especially in immune cells. ITAM’s tyrosine residues are phosphorylated by Src (sarcoma) kinases, with the consequent recruitment of Syk kinases (spleen tyrosine kinases), which then phosphorylate other substrates involved in cell proliferation and differentiation. It is interesting to note that ITAMs are encoded also by well-known oncogenic viruses, such as EBV and BLV. The ITAM associated to Env was shown able to initiate malignant transformation of mammary epithelial cells: depolarization of mammary acini, transformation of the mammary epithelial phenotype, and transformation of human mammary epithelial cells. It follows that *env* has the qualities to be defined as oncogene [76,77].

#### 3.7.3. Transcription

For its transcription the provirus needs a DNA sequence named HRE, hormone response element, located in position −202 and −59 upstream of the start site of transcription of the LTR region (long terminal repeat), which is activated by glucocorticoids, progestins, androgens, and prolactin. Glucocorticoids have been used for decades to stimulate MMTV production in cell culture [78,79].

Estrogens play a different role. The provirus integrates more easily in the DNA of the host cells during cell division. Estrogens increase the proliferative rhythm of the mammary gland epithelium. Moreover, estrogens are indispensable for the progression of mammary tumors, once the neoplastic process is initiated.

### 3.8. Mouse Strains and Their Tumors

Mouse strains hosting MMTV are numerous. As a consequence of the viral infection, they can have a different incidence of mammary tumors. For instance, C3H, RIII, and GR mice have a high incidence, with tumors arising “early” (before one year of age), while C57BL and BALB/c have a low incidence, with tumors arising “late” (after one year) [80].

MMTVs of different mouse strains can be identified with a letter, such as S (standard, the classic Bittner virus) in C3H and RIII, P (plaque, see below) in GR, L (low incidence) in C3HfC57BL, O (old retired breeders) in BALB/c, X (radiation induced), W (wild), etc. [81].

MMTVs can be also identified by the name of the strain studied, such as MMTV-C3H, MMTV-RIII and so on [also C3H-MMTV, MMTV/C3H, MMTV(C3H) according to the authors] [80].

Tumors of the mouse mammary gland have been extensively studied and classified. Two main types are the “adenocarcinoma type A” typical of C3H mice and the “adenocarcinoma type B” typical of RIII mice. According to Thelma Brumfield Dunn, type A tumors have a “fine uniform acinar structure” with small uniform acini, whereas type B “represent a diversified, multiform group in which the tumor is clearly of glandular epithelial origin, but in which there are no predominant features”, with acinar structures, solid areas, irregular glands, cysts filled with blood or clear fluid, intracystic papillary projections etc. [82].

The model of MMTV mouse mammary tumors was crucial for the development of the concept of tumor progression and preneoplastic/preinvasive lesions, as a mainstay of modern oncology [83]. In fact, two types of these lesions have been identified: the hyperplastic nodules (later HAN, hyperplastic alveolar nodules) and the plaques. Interestingly, there is a relationship among type of tumor, type of “precancerous lesions”, and mouse strain: C3H mice develop HAN and type A tumors, RIII mice develop plaques and type B tumors. Whereas HAN are hyperplastic lesion similar to the proliferative lesions of human breast, plaques are benign tumors in which foci of adenocarcinoma appear later and conquer the entire gland.

### 3.9. How Many Types of MMTV?

The question, “How Many Types of MMTV are there?”, was asked several decades ago, but a complete answer is still missing. A difference of about 20% has been reported in the viral genome between high-incidence and low-incidence mouse strains. Conversely, no sound difference was demonstrated among the high tumor incidence strains [80].

However, an experiment of 60 years ago unveiled the existence of types of MMTV with relevant biological dissimilarities [84].

Francesco Squartini was intrigued by the different characteristics of C3H and RIII mammary tumors. The C3H tumors have HAN as preneoplastic lesions, are type A adenocarcinoma, are hormone independent, and are highly metastatic. The RIII tumors have plaques as precancerous lesions, are type B adenocarcinoma, are hormone dependent, and are poorly metastatic. The question asked was as follows: Are these morphological and biological differences, which are so evident in the mammary carcinogenesis between C3H and RII mice due to the different genetic constitution of the animals, or to relevant differences between MMTV/C3H and MMTV/RIII viruses?

The answer was obtained by a “simple” experiment aimed to cancel the influence of the genetic characters of the host: to study the tumors induced by the two different virus strains when introduced in the same mouse strain. Two new mouse strains were created by fostering BALB/c mice by C3H or RIII mice; actually, the BALB/cfC3H had been already established, whereas the BALB/cfRIII was brand new. BALB/cfC3H animals developed mammary tumors identical to those of the C3H strain, whereas BALB/cfRIII animals developed tumors identical to those of the RIII strain. “These results indicate that the tumor characters may be transferred from one strain to another by means of foster-nursing”.

The intentionally soft conclusion of Squartini means that different MMTV strains exist, able to drive different carcinogenetic pathways.

### 3.10. MMTV Can Infect Adult Mice by an Alternative Way of Entry: The Nasal-Associated Lymphoid Tissue

As seen above, in the newborn mouse MMTV enters the small intestine at the level of Peyer’s plaques, where an intimate interaction with the immune system takes place.

In 1997, Swiss scientists were successful in infecting adult mice with mouse milk containing the viral particles [85]. They demonstrated that, once injected into the nose, MMTV infects the nasal-associated lymphoid tissue (NALT) with modalities similar to those observed in the Peyer’s plaques.

Hence MMTV can enter an organism even from sites different from the small intestine, provided that well represented lymphatic structures are present.

These findings are quite relevant in the formulation of hypotheses for the inter-human transmission of HBRV.

### 3.11. Not Only Mammary Tumors

MMTV has a pleiotropic role, in that it is able to induce in mouse tumors other than the mammary ones.

The BALB/cf/Cd mouse has a 70% incidence of spontaneous kidney adenocarcinomas, which show viral particles, the presence of several copies of integrated exogenous MMTV, and an *Int* common to mammary and kidney tumors [86,87].

MMTV is able to induce also T-cell lymphoma/leukemia (MMTV variant type B leukemogenic virus—TBLV) and B-cell lymphoma [88,89,90]. This effect can be secondary to the intimate relationships that the virus has with the immune system, essential for the infection. In 1988, Judith Ball et al. demonstrated the presence of alterations in the U3 region of the LTR of the type B thymotropic DMBA-LV retrovirus, which are able to induce T-cell lymphomas [91]. In 1993, Walter Gunzburg et al. identified in the MMTV U3 region a novel promoter (P2), considered the major promoter used for expression of Sag in B lymphocytes, with consequent amplification of B and T cells [68]. In 2000, Brian Salmons et al. demonstrated that Sag expression is driven independently by both MMTV promoters (P1 and P2) [92].

### 3.12. Endogenous MMTV

The previous pages dealt with the exogenous form of MMTV, a viral particle that buds on cell surface and is transmitted horizontally via milk. However, MMTV exists also as an endogenous form, a particle that usually does not bud and is transmitted vertically by germ cells according to the Mendelian inheritance. The exogenous virus is indicated with the acronym MMTV, while *Mtv* indicates the endogenous virus. *Mtv* is an ERV (Endogenous Retrovirus). ERVs are retrotransposons.

#### 3.12.1. Transposons, Retrotransposons, ERVs

Transposons or transposable elements (TEs) are DNA sequences that are able to move within the genome. The 1983 Nobel prize for Physiology or Medicine was awarded to Barbara McClintock for her discovery of mobile genetic elements [93]. Transposons can be of two types: retrotransposons, copied by an RNA segment by a reverse-transcriptase, and DNA transposons.

Retrotransposons are subdivided according to the presence or absence of LTRs (long terminal repeats) flanking a central coding region. Non-LTR groups are LINEs (long-interspersed nucleotide elements) and SINEs (short-interspersed nucleotide elements). LTR-retrotransposons are the endogenous retroviruses (ERVs).

ERVs are sequences of exogenous viruses inserted into the host genome millions of years ago that have remained with no functions (living fossils) or with functions mainly regulatory. Three classes of ERVs can be recognized on the base of their similarity with exogenous retroviruses: class I: resembles gamma and epsilon retroviruses; class II: alpha, beta, and delta retroviruses; class III: spumaviruses. Class II includes *Mtv*, MusD/ETn (early transposon) family, and IAP (intracisternal A particles) [94,95,96,97,98].

ERVs usually do not own the *env* gene, in that they do not have the envelope.

In the previous chapters, it was clarified that MMTV is the B particle, formed by the budding at the cell membrane of an intracytoplasmic A particle, its immature form. However, A particles are the morphological expression also of the ERVs, IAP in particular, with the difference that they accumulate in the cytoplasmic cisternae and do not form infectious particles. Two facts explain why IAP are unable to become B particles and to infect: (a) IAP have a long LTR with *gag*, *pro*, and *pol* genes, whereas *env* is missing; (b) the matrix domain (MA) of the Gag polyprotein is lacking in its myristoylation signal, necessary for anchoring the lipid molecules of the cell membrane, preventing Gag from exert its role in assembling the mature virions.

However, it is well known that mice hosting ERVs can form XRVs (exogenous retroviruses), as in the GR mouse strain, in which the virus can be transmitted by milk. When an endogenous virus becomes infectious and forms B particles, IAP are, however, present and indistinguishable from those of strictly non-infectious endogenous MMTV, but with a molecular difference that easily explains their new behavior: they have an *env* gene. No longer IAP, but IAPE, “intracisternal A-particle-related elements coding for *env*elope”. There are data in favor of the fact that IAPs descend from IAPEs [99,100,101].

ERVs can express an oncogenetic action through at least a couple of mechanisms, such as insertional mutagenesis or the production of proteins that play a role in carcinogenesis [102,103,104].

**Part III: Human Mammary Tumor Virus—HMTV**

## 4. The Human Mammary Tumor Virus

### 4.1. MMTV in Human Milk

On February 1971, Nature titles in *News and Views* [105] “Human breast cancer virus?”, reporting that “Moore and his colleagues [106] have detected this particle (read MMTV) in seven out of one hundred and fifty-six samples of milk from Philadelphia women with no history of breast cancer in their family, in six out ten milks from American women whose near female relatives had the disease and in eighteen out forty-six milks from Parsi mothers living in Bombay”, respectively, 4.48%, 60%, and 39%. Parsi women were an object of interest because of their high breast-cancer incidence. These results would deserve a careful interpretation on the basis of the wide amount of knowledge available today, hereditary cancers included. However, this paper reports for the first time the presence in human milk of particles with a very convincing B particle morphology and their association with breast cancer.

At the same time (1970), the reverse transcriptase was discovered by David Baltimore [107] and Howard Martin Temin [108]. This novel finding was immediately applied to MMTV. Jeffrey Schlom, Sol Spiegelman, and Dan H. Moore demonstrated that human milk containing B particle contains also the reverse transcriptase, while milk without particles lacked the enzyme [109]. Then, Schlom and Spiegelman described a simultaneous assay of the reverse transcriptase and high molecular weight RNA unique to oncogenic RNA viruses [110] and, with other colleagues, were able to quantitate the viruses in human milk by hybridization of the polyadenylic acid regions in their 60S to 70S RNA [111]. Moreover, they isolated from human milk viral cores (nucleoids) that have a density of 1.26–1.27 g/mL and contain 60–70S RNA and RNA instructed DNA polymerase, features diagnostic of retroviruses [112].

Other laboratories were not able to morphologically identify viral particles in human milk; however, they highlighted the relevance of the presence of reverse transcriptase [113]. Quite interestingly, a destructive effect exerted by human milk on MMTV particles was demonstrated, affecting all three measures of viral integrity, i.e., morphology, infectivity, and reverse transcriptase [114], which can justify the different results found.

As described above, the early 1970s were a period full of enthusiasm for the possibility of a viral etiology of human breast cancer and of discoveries in that direction. The driving idea was that the human model could copy the murine model, with MMTV present in milk and transmitted to newborns. Results of morphological and biological studies on milk, even if not unanimous, supported this idea. Unfortunately, epidemiology did not: “the weight of epidemiologic data has not been consistent with milk transmission of human breast cancer” and “no association between breast feeding and breast cancer risk in offspring was found”. Yet, “there is little doubt of a definite genetic component to human breast cancer. The increased familial risk to this disease seems to be associated with an altered state of hormone excretion or metabolism” [115]. All these three points were shown correct in the following years: (a) the search for MMTV in the milk was essentially abandoned because of the lack of convincing results [116]; (b) the hereditary/familial cancer became a reality; (c) the absolute relevance of estrogens in breast cancer progression was demonstrated, with its therapeutic implications. However, the fact that in humans the transmission of MMTV by breast feeding is highly unlikely does not exclude the presence of MMTV in milk.

### 4.2. MMTV in Human Cancer

#### 4.2.1. Molecular Biology and Immunohistochemistry

Because of the discouraging results given by searching the virus in human milk, scientists began to focus on breast-cancer tissues.

In 1972, Sol Spiegelman, using molecular hybridization, demonstrated the presence of RNA homologous to MMTV RNA in 66% of 29 infiltrating breast cancers, with normal breast tissues, benign breast lesions, and non-breast tumors all negative [117]. In the same year, they reported the identification of a 70S-RNA-instructed DNA polymerase in extracts of 30 out of 38 breast carcinomas, with a percentage of 79%; ten non-malignant lesions were negative [118]. They also showed that “the enzyme and its RNA template are localized in a particle possessing a density characteristic of the RNA tumor viruses and that the DNA synthesized hybridizes specifically to the RNA of MMTV” [118]

Biochemical evidence is also provided for the presence of an RNA tumor virus in purified fractions of human malignant breast tumors; these particles are similar to the subviral particulate of MMTV isolated from murine mammary tumors; furthermore, particles exhibit features diagnostic of cores and ribonucleoproteins of oncornaviruses [119].

Then, the same laboratory adopted the newly developed immunohistochemical approach to the study of proteins and identified, in paraffin sections of human breast cancer, an antigen, immunologically related to a group-specific antigen of the MMTV, the gp52, a 52,000-dalton glycoprotein.

#### 4.2.2. The gp52 Glycoprotein

As seen before, the *Env* gene, which is a main gene of MMTV, encodes for a gp70 polyprotein that gives origin to a couple of subunits, SU (surface) and TM (transmembrane). The SU is a gp52 glycoprotein.

For the above reasons, Spiegelman et al. decided to study the immunohistochemical expression of gp52 in human samples. The protein was demonstrated in 39% of 131 infiltrating breast cancers, whereas all 119 samples of normal breast and benign breast lesions were found negative; moreover, 99 non-breast tumors and eight cystosarcoma phyllodes of the breast were all negative; interestingly, some cases of negative benign lesions were coincidental with a positive breast carcinoma. In an extended series of 376 cases, the percentage of positive cases arose to 45.5% [120,121]. Interestingly, they were able to localize gp52 protein (using antibodies specific or against the MMTV) in axillary lymph nodes before the discovery of the primary breast tumor, which then proved to be gp52 positive [122].

Finally, they demonstrated a positivity for gp52 of the particles released from the human T47D breast-carcinoma cell line, which have characteristics of retroviruses [123].

## 5. The Env Sequence, the HMTV, and the Breast Carcinoma: The New York Laboratory

As described in the first part of this article, the *Env* gene is one of the main genes of MMTV, and is very important for its life cycle and, therefore, of high interest in the search for MMTV in human tissues. It has been well studied since the 1970s, and received more attention in the first decade of this century after the demonstration of its direct role in carcinogenesis. In fact, it encodes an ITAM (immunoreceptor tyrosine-based activation motif), which is able to trigger transformation in murine and human mammary epithelial cells and to function in non-hematopoietic cells as potent oncoproteins by scaffolding downstream mediators [76,124].

### 5.1. Env Sequences in Human Breast Carcinoma

A renewed and strengthened interest towards a possible human MMTV as a cause of breast cancer appeared after about twenty years after the studies on gp52 protein, when in New York City, Beatriz Pogo et al. decided to focus on the presence of *env* gene in human breast cancers. In 1995, they were able to detect a 660-bp MMTV *env* gene-like sequences in a large part (38.5%) of 314 breast cancer samples and in two human breast-cancer cell lines. The sequence was selected on the basis of its very high homology to MMTV (95–99%) and of its very low homology to human endogenous retroviruses (<15% when compared to the endogenous provirus HERV-K10) or to all known viral and human genes (≤18%; GenBank, GCG programs). The 660-bp sequence does not seem to belong to an endogenous retrovirus, as demonstrated also by the fact that normal organs and non-breast cancers were all negative, as well as blood lymphocytes from positive breast cancer patients and breast tissues homolateral to cancer. Additionally, a 250-bp sequence within the 660-bp stretch was used for the analysis of paraffin tissues and was found present in 40% of breast cancer samples. Two human breast-cancer cell lines (MCF-7 and ED) were shown to contain the 660-bp sequence, while four other cell lines were positive only for the 250-bp sequence (T47D, BT474, BT20, and MDA MB 231) [125]. The high homology of the *env* sequence to MMTV and its low homology to endogenous retroviruses were demonstrated further in a following paper [126].

### 5.2. Env Sequence Expression

The 660-bp sequence was found expressed in 66% of *env*+ breast cancers and the 256-bp sequence in all *env*+ cancers, while they were absent in *env*− cases. The 660-bp sequence was 98% homologous to the *env* gene of the MMTV [127].

### 5.3. MMTV Provirus

Then, a whole proviral structure from each of two human breast carcinomas that were *env* positive was amplified, in particular LTR-*gag*, *gag-pol,* and *pol-env* segments. The 9.9-kb provirus is 95% homologous to MMTV but only 57% to human endogenous retrovirus K10 in 3.5 kb.

*Gag* and *pol* showed a higher homology to HERV-K10. PCR approaches appear never to have been confirmed using regions of low homology in the *gag* and *pol* genes. It can be hypothesized that there should be regions (such as the dUTPase or part of pol) that should be homologous enough to allow confirmation that human breast cancer contain HMTV (e.g., in fluorescence in situ hybridization with *gag/pol* primers in *env*-positive breast cancers and breast cancer cell line but not in chromosomes from normal breast cells).

The provirus displays typical features of a replication competent virus, plus the open reading frame for the superantigen and the glucocorticoid responsive element. Fluorescence in situ hybridization with a 2.7-kb *env*-LTR sequence of an *env*-positive breast cancer cell line revealed that the sequence is inserted in several chromosomes but not in chromosomes from normal breast cells [128].

### 5.4. MMTV-Like LTR and LTR-Env Gene Sequences

At the same time, MMTV-like LTRs were detected in human breast cancer. A 630-bp LTR sequence, amplified by PCR using primers LTR5 and LTR3, was identified in 41.5% of 65 human breast cancers, with normal breast tissues negative. A larger 1.2 kb LTR fragment was also amplified with high homology to MMTV. Finally, a 1.6 kb fragment containing *env* and LTR sequences was amplified, cloned and sequenced from breast-cancer DNA. The human LTRs were highly homologous to MMTV and contain enhancer and promoter elements, the glucocorticoid responsive element (GRE) and the super-antigen (Sag) sequences. The presence of functional sequences implies involvement in transcriptional regulation, whereas the presence of an *env*-LTR sequence indicates contiguity within the genome of a potential provirus. Their presence in breast-cancer DNA, but not in normal tissue, suggests an exogenous origin [129].

### 5.5. The Superantigen (Sag)

MMTV-like *sag* sequences were amplified and sequenced from 10 human breast cancers and found highly homologous to those of MMTV. Moreover, the LTR Sag was shown able to stimulate human T lymphocytes inducing them to proliferate and to secrete cytokines, obtaining the same response that occurs in the mouse [130].

### 5.6. The Interferon Signature

Stimulated by a paper of Uri Einav et al., reporting the expression of an interferon-related signature in breast and ovarian cancer [131], researchers compared the expression profile of two sublines of MCF-7 cells, one containing the MMTV-like sequences and the other one lacking them. Among 27 genes upregulated in the *env*+ cells, 7 were interferon-related, 5 TNF-connected, and 2 TGFβ-related. The authors concluded that MMTV sequences stimulate a chronic inflammatory response, expected in a viral infection and in line with the intense relationship between MMTV and the immune system in the mouse [132]. B and T lymphocytes are indispensable for establishing the infection and for spreading the virus in the host, as mentioned before.

### 5.7. MMTV Particles in Human Cells

As already seen, in the previous years, many attempts were undertaken to identify MMTV particles in human cancer cells, without conclusive results. Pogo’s laboratory identified them in primary cultures of cells obtained by pleural effusions of patients with metastatic breast cancer. Particles showed ultrastructural characteristics compatible with MMTV. Env protein was expressed and reverse transcriptase activity was determined. cDNA sequences from virion DNA were synthesized, amplified, and sequenced; all the virion genes were detected, and 70% of the virion RNA was sequenced. The sequence homology with MMTV and human MTV proviruses were 85% and 95%, respectively. A 52 kDa protein was detected in tumor cells [133].

### 5.8. MMTV Proteins in Human Cells

Envelope and capsid viral proteins were detected in primary cultures of human breast cancer cells containing human MTV sequences by Western blot and FACS using a panel of antibodies [134].

The expression of HMTV envelope (Env) and capsid (Ca) was detected in 10 primary cultures of human breast cancer containing HMTV sequences (MSSM) by Western blot and fluorescence activated cell sorting (FACS), using a panel of antibodies against HMTV Env, HBRV Env and Ca and the MMTV Env Gp36 and Ca P27 proteins. All the antibodies detected MMTV proteins. Approximately 13% of the MSSM cells showed HMTV protein expression by FACS analysis.

### 5.9. The HMTV—Human Mammary Tumor Virus

On the basis of the results obtained in her laboratory, Beatriz Pogo introduced the term Human Mammary Tumor Virus (HMTV), to express in a synthetic but exhaustive way her convincement of the existence of a human homolog of MMTV [128].

## 6. MMTV/HMTV Is Significantly Present All over the World

Data provided by Pogo’s laboratory motivated many other groups to investigate the presence of *env* sequences in human breast cancers. Most of the laboratories had positive results, while others could not find a correlation. Almost all studies analyzed the original *env* sequences. Table 1 summarizes all the published studies [125,129,135,136,137,138,139,140,141,142,143,144,145,146,147,148,149,150,151,152,153,154,155,156,157,158,159,160,161,162,163,164,165,166,167,168,169,170,171,172,173,174,175,176,177,178,179], ordering them by continent and by country (20 in total). The first continent to be mentioned is North America because of Pogo’s first paper, and it proceeds clockwise and north-south.

The table lists 46 articles, with a total of 51 patient populations. MMTV sequences are present in 42 populations (82%), while absent in 9 (18%). The total number of cases studied is 5015, with 1320 positive cases, accounting for a 26%. The percentage of positivity among North America, South America, Europe, and Asia is substantially the same (18–28%, similar to the global value). The percentage doubles in North Africa (40%) and Australia (40%).

The studies were conducted on frozen material or on FFPE tissues, with the majority on the latter. The laboratory procedures seem substantially adequate; however, it is hard to go into details. It is impossible to obtain clear information about histotypes and hereditary tumors.

Some papers report positive cases also in normal breast tissues, in benign breast lesions, in blood, and in human cancer cell cultures.

There is one and only one message that is possible to get from the Table 1: MMTV is significantly present in breast cancer all over the world.

Some of the authors of the “negative” papers suggest that the “positive” data could be a consequence of a contamination with endogenous viral sequences or with plasmids or amplimers, actually denying the viral hypothesis for BC. Others suggest that the absence of viral sequence could be due to a concentration too low for being detected or to a different geographic and ethnic distribution of the virus. A possible explanation that can be considered “prophetical” (2003) is that “the agent may be a still unknown human retrovirus” [144]. Moreover, “our findings imply that geographic and ethnic variations might play a significant role in MMTV-like virus infection and its role in breast cancer development” [163]. These points will be later discussed in detail.

The 2011 article of Daniel Park et al. [180], with only negative results, was not included in the Table 1 because its population consists only of hereditary tumors; the absence of a viral involvement in hereditary neoplasia was shown in 2019 [150].

## 7. HMTV Is Able to Infect Human Cells: The Vienna Laboratory

### 7.1. TfR1—The Transferrin Receptor 1

Twenty years ago, the laboratory of Susan Ross showed that the mouse TfR1 is the entry receptor for MMTV [58]. Then, the same authors demonstrated that MMTV does not use human TfR1 to infect cells, even if it is able to bind both the murine and the human receptors [181]. These data were often used to support positions against the viral etiology of BC.

### 7.2. HMTV Can Infect Human Cells

In 2005, in Vienna, Stanislav Indik, Walter Günzburg, Brian Salmons, and Francoise Rouault demonstrated that MMTV is able to infect human normal (Hs578T) and cancerous (T47D and MCF7) cells [182]. Then, they showed the rapid spread of MMTV in Hs578T cells, which leads to the infection of the entire cell population in culture [183]. The replication of the virus was monitored by quantitative PCR and RT-PCR and by immunofluorescence and B particles were released. Replication of the virus was blocked by inhibition of reverse transcription. Then, different MMTV strains (C3H and GR) were shown to be able to infect human cells [184].

### 7.3. HMTV Does Not Use TfR1 to Infect Human Cells

Finally, Indik et al., 7 years ago, already had demonstrated that MMTV is capable of infecting cells in the absence of TrF1. In fact, the viral Env G42E mutation enhances the interaction virus/receptor on the cell surface and confers to the virus a greater infectivity. The mutation consists in a substitution of glutamic acid in place of glycine at position 42 of SU. Authors concluded that the transferrin receptor 1 is not essential for infection and that a different cell surface molecule mediates the MMTV entry into human cells [185].

## 8. HMTV Is Involved in Almost All Sporadic Breast Carcinomas: The Pisa Laboratory

### 8.1. Stringent Laboratory Procedures Guarantee the Quality of the Result

In 2006, the author, stimulated from Pogo’s results on human carcinomas [125] and from the papers reporting no association between MMTV and human BCs, decided to involve his laboratory in this new field of study. The aim was to develop a strategy able to avoid any possible contamination with murine material by combining a highly sensitive and specific PCR-based method (fluorescent nested-PCR), also simple and reproducible, with an automatic laser-assisted microdissection procedure. Microdissection allows researchers not only to obtain a pure cell population, but also to enrich it, i.e., to have, through serial sections of the same fragment, a number of cells more than sufficient for an optimal quantity of nucleic acids. In this first experiment, only frozen samples were used. The result of 33% of BCs positive for HMTV [148] confirmed with no doubt Pogo’s data. In the following studies, FFPE were used, keeping fluorescent PCR and laser microdissection.

### 8.2. HMTV Is Present in the Vast Majority of Sporadic Breast Carcinomas

In a previous chapter, the concepts of cancer progression and of preinvasive lesion were discussed.

Applying the methodology illustrated above, *env* sequences were investigated in the various types of preinvasive lesions. As expected, HMTV is well expressed since the beginning of the nuclear modifications, with a 27% of positive cases in ADH (atypical ductal hyperplasia). Quite surprisingly and interestingly, DCIS (ductal carcinoma in situ) doubles the positivity of invasive tumors, 82% vs. 35%. The dramatic decrease in positive cases in invasive tumors is paralleled by the even dramatic decrease in the viral load analyzed by CISH (chromogenic in situ hybridization). The virus is present also in normal glandular structures proximal to invasive carcinomas (19%), while normal epithelia at distant sites are always negative [149].

The interpretation of these results is made easier bearing in mind that gene mutations induced by MMTV integration are initiating events [73], not involved in the process of cancer progression: (a) HMTV infects the epithelial cells of the mammary gland and transforms part of them (initiation); (b) the entire population proliferates (hyperplasia); (c) the transformed cells show atypical nuclei (atypical hyperplasia); (d) the entire population becomes neoplastic (carcinoma in situ); (e) the molecular pathways activated by the viral insertions give rise to the events typical of progression, with the result of an invasive carcinoma.

The halving of the positive cases in invasive lesions when compared to DCIS can be explained by the fact that the virus is no longer necessary and can be lost [149]. In fact, IDCs are characterized by a high level of chromosomal rearrangement. An intervention of the immune system is also possible.

### 8.3. Hereditary Breast Carcinomas Do Not Need a Viral Etiology

As detailed in the first part of this article, HBCs recognize a specific genetic etiology, based on the hereditary transmission of highly penetrant pathogenic mutations affecting a TSG. As a consequence, it was hypothesized that HBCs do not need an exogenous etiological agent, such as HMTV, for their development.

A study on the presence of viral sequences in two groups of sporadic and hereditary breast tumors confirmed the hypothesis: HBCs have a very low percentage of cases positive for HMTV, only 4% (2/47), in comparison to a 30% (17/56) of sporadic carcinomas. All patients with HBCs hosted a BRCA germline mutation [150].

Interestingly, this study well supports the reliability of the data obtained by FFPE tissues. In addition to the usual laboratory precautions, all the paraffin blocks (HBCs and SBCs) were from the same archive and were processed in the same laboratory, with the same equipment and reagents; moreover, the laboratory did not host mice nor murine cell cultures. Such a significant difference (30% vs. 4%) cannot be due to a contamination with murine material, unless of an improbable “selective” contamination.

These data on HBC also allow researchers to view in a different light Daniel J. Park et al.’s 2011 paper [180] reporting the absence of MMTV in a series of 42 invasive breast carcinomas, which suggests that the positive cases reported in the literature were all due to PCR contamination. Interestingly, a careful reading of Park’s article shows that all the patients were enrolled in the Australian Breast Cancer Family Study, and all of them received a diagnosis of cancer before the age of 40. It is reasonable to hypothesize that all these cases were HBC and by default MMTV negative. Moreover, Beatriz Pogo [186] criticized the laboratory methods adopted by Park et al.

### 8.4. HMTV in Saliva and the Paleoanthropological Study

These studies will be described in Part IV.

## 9. The P14, A MMTV Signal Peptide–A Possible Tool for Vaccination Strategies: The Laboratory of Jerusalem

Jacob Hochman et al. identified a MMTV p14 protein in tumorigenic and nontumorigenic cells of murine T-cell lymphomas [187]. This protein, an *Env*-derived peptide, has a nucleolar localization and interacts with the nucleolar protein B23 (nucleophosmin, numatrin). B23 is associated with other retroviral proteins, such as Rev and Rex [188].

The authors developed antibodies against recombinant p14, which revealed it was useful to localize it in the nucleoli of murine mammary carcinomas and human BC [189]. Actually, p14 antibodies are a reliable and useful tool for immunohistochemical analysis of MMTV in human cancer [190].

Notably, p14, identified as the signal peptide of MMTV *Env*, was shown to be a phosphoprotein with two phosphorylation sites, at serine 18 and 65. p14 behaves as a tumor modulator: a Ser65Ala mutation is associated with impaired tumorigenicity, whereas Ser18Ala mutation is associated with enhanced tumorigenicity [191].

Finally, Hochman et al. showed p14 expression on the cell surface of both murine and human cells containing MMTV. Since p14 is immunogenic, they propose it as target for vaccination strategies [192].

## 10. The Point of View of an Epidemiologist: The Laboratory of Sidney

In the last 20 years, James Sutherland Lawson (Jim to his friends) et al., put an amazing energy in the transmission to the scientific community of their conviction that viruses play a major role in human diseases: breast cancer, prostate cancer, atherosclerosis. MMTV occupies a major place in their interests and some of their many results deserve a special mention: the presence of MMTV in breast tissues years before the development of a carcinoma [193], the resemblance between murine and human breast tumors [178], the high percentage of BC cases positive for MMTV in an Australian population [174].

Details about Jim’s work are outlined in the article co-authored with Wendy K. Glenn published in this same issue of Viruses [194].

Jim also wrote two delightful books, one on his adventurous life, in which MMTV plays an important role [195], and one on the history of MMTV, published this month [196].

## 11. HMTV in Human Neoplasia Other Than Breast Carcinoma

In humans, an association has been found between MMTV and non-breast tumors: non-Hodgkin lymphoma (NHL), tumors influenced by hormones, and lung cancer.

### 11.1. Non-Hodgkin Lymphoma (NHL)

The association between BC and NHL has been described during the last twenty years [197,198]. Quite interestingly, HMTV have been detected in both types of tumors from the same patients [135,138]. Moreover, MMTV induces both mammary tumors and lymphomas in mice and has strict relationships with the immune system for infecting the host and for spreading the infection.

### 11.2. Tumors Influenced by Hormones

HMTV has been detected in carcinomas of endometrium (10%), ovary (16%), and prostate (36%) [199,200]. This result is in some way expected, considering that the carcinogenesis of all these tissues, as for the breast, is strongly influenced by estrogens and the MMTV LTR promoter is induced by steroids.

### 11.3. Lung Cancer

HMTV has been detected in a few cases of lung cancer. Unfortunately, not sufficient information about the specimens is provided [201].

Taken together, these observations (lung cancer apart) are a solid suggestion for a viral role of HMTV in human carcinogenesis, not limited to the breast, as is also observed for MMTV in mice.

## 12. HMTV and the Human Primary Biliary Cholangitis: The Laboratory of Edmonton

The story begins in 1998, when Andrew Mason et al. showed that many patients with primary biliary cirrhosis (PBC) had antibody reactivity to proteins that share antigenic determinants with HIV-1 p24 or HIAP (human intracisternal A particles) [202]. Having in mind a 1990 paper [203] reporting human IAP in lymphoblastoid cells exposed to homogenates of salivary tissue from patients with Sjogren’s syndrome, the same authors identified retroviral B particles from PBC biliary epithelial cells of patients with PBC and cloned exogenous retroviral sequences from the same patients. The name “human betaretrovirus” was introduced [204].

Mason’s scientific work is of special relevance in the research of a human betaretrovirus. An important component of this work was to link human betaretrovirus with autoimmunity by detecting infection in cells displaying upregulated mitochondrial autoantigen expression [204]. A new aspect of viral pathogenesis was described in co-culture transmission studies showing PBC lymph nodes and pure MMTV isolates could trigger the PBC disease expression of mitochondrial antigens in healthy biliary epithelial cells [204]. Following is a list of his major accomplishments.

Using perihepatic lymph node DNA, in 2004, human betaretrovirus nucleic acid sequences were cloned from patients with PBC [205]. They share marked homology with MMTV and human breast cancer-derived retrovirus sequences. In 2007, a reverse transcriptase activity was demonstrated in patients with primary biliary cirrhosis and other autoimmune liver disorders [206].

Then, a correlation between MMTV infection and the production of AMA (anti-mitochondrial antibodies) was shown in many mouse models of primary biliary cirrhosis, where MMTV proteins were localized to cells displaying upregulated mitochondrial autoantigen expression [207]. Antiviral therapy was successful in treating the NOD.c3c4 mouse model of PBC with a reduction in serum liver-enzyme levels, attenuation of cholangitis and decreased MMTV levels in the liver [208].

In 2015, the human betaretrovirus was found frequently integrated in biliary epithelium of patients with PBC, autoimmune hepatitis and other idiopathic liver disease [209]

In 2020, human betaretrovirus was produced, purified and characterized and antibodies against the viral surface protein (Su) were detected in patients with breast cancer or liver disease [210].

In the meantime, several papers on antiviral therapies in patients with primary biliary cholangitis were published reporting significant improvements in liver biochemistry and histology [211,212,213,214,215].

An important result is illustrated in an article published in this same issue of Viruses: this year they were able to isolate a human betaretrovirus from patients with primary biliary cholangitis [74].

## 13. HERVs—Human Endogenous Retroviruses

The search in humans for exogenous retroviruses must take into account the existence of HERVs. In fact, their presence can cause false positive results. For a better understanding of the issue, the following notes can be useful. Murine ERVs have been already discussed.

Millions of years ago, unknown retroviruses used to infect the somatic cells of vertebrates. Sometimes, germ cells were infected and viruses could become stably integrated in their genome, acquiring the ability to be transmitted to the next generation (Mendelian inheritance). These retro-elements (or transposable elements) are called endogenous retroviruses (ERVs). In humans, they are called HERVs (human endogenous retroviruses) and comprises about the 8% of the genome. A complete HERV is made of four viral genes (*env*, *gag*, *pol*, *pro*) flanked by LTRs [216].

The accumulation of mutations, insertions, and deletions, occurring over approximately 30 million years of evolution caused profound changes in the structure and function of HERVs, with the complete loss of the coding region with a homologous recombination of the two flanking LTRs. In fact, solitary LTRs are the most common evidence of HERVs [217].

To date, it is not known whether HERVs are able to replicate. However, some of them can code for proteins and a few can produce viral particles. An example of HERVs involvement in physiological processes, is Syncytin-1, coded by an HERV-W provirus, involved in placental development. Quite interestingly, LTRs can play several regulatory functions, acting as promoters, enhancers, and repressors, etc. A regulatory role of the immune system has been described, as well as a role in oncogenic signaling pathways [216]. However, despite the abundant scientific production that links HERVs to human disease of various types (schizophrenia, diabetes, autoimmune diseases, cancer, etc.), there are no conclusive data about their possible pathological role [218]. However, concerning cancer, HERVs could induce carcinogenesis activating oncogenes or inhibiting suppressor genes. Moreover, their positive or negative effects on immunity could, in some way, contribute to carcinogenesis [219].

To classify HERVs is a complex task, far from its conclusion. However, HERVs are subdivided in three classes: I (gamma- and epsilon-like), II (beta-like), and III (spuma-like), on the basis of their similarity to exogenous retroviruses. The HML supergroup of class II (human MMTV-like) arouses a special interest, for its relationships with MMTV, and, as consequence, with a well-known murine model of oncogenesis [217].

Quite interestingly, the production of MMTV B-like particles were described in human cells and considered of endogenous origin [220]; moreover, HERV-K113 was found capable to produce particles with a retroviral morphology [221].

## 14. The Leitmotiv of Contamination: No Evidence

The research of a viral etiology for human breast cancer has been accompanied since its beginning by a doubtful school of thought, according to which the presence of MMTV sequences in human breast carcinomas is due to contamination with murine material. It must be emphasized that some of these laboratories do not seem familiar with the biology of the MMTV and/or of the human breast carcinoma. Moreover, some of these papers were published even years before clarifying discoveries.

Some of these papers [222,223,224,225,226,227] have been cited with some frequency throughout the years, usually uncritically. According to them, the viral hypothesis of human BC cannot be supported on the basis of the following observations, only a few of which are concerned with contamination.

### 14.1. Contamination

1. Results showing a correlation between MMTV/HMTV/HBRV are false positive caused by contamination with murine DNA containing integrated MMTV DNA.
2. Rodent DNA is present throughout building walls and ventilation systems.
3. Negative data are consistent with the majority of papers on this topic, while only a few labs describe MMTV positive human breast-cancer specimens.

The present article summarizes the numerous studies demonstrating that the viral sequences investigated are exogenous and human, using appropriate laboratory procedures. In particular, the use of frozen tissue, of the laser microdissection for the selection of the cell population and its enrichment, and of the fluorescent PCR represents a quality assurance [148].

The studies carried out in Pisa were performed in laboratories that did not host animal facilities, nor viral cultures, and neither murine cell cultures; the author believes that at least the largest part of the laboratories in which similar studies were conducted was in the same condition.

Statement n. 3 is not supported by the analysis made in this article, as it appears by the reading of the Table 1.

Moreover, some facts cannot be interpreted due to contamination:

- In the same laboratory (Pisa), with the same equipment and reagents, the percentage of MMTV positive cases remains constant in the years [148,149,150]: (a) in 2006, 33%, (b) in 2011, 35%, (c) in 2019, 30.3%.
- Again, in Pisa, the positivity was of 4% in hereditary tumors against 30% in sporadic ones; all the FFPE tissue came from the same archives and was processed in the same laboratory with the same equipment and reagents [150]. The striking difference cannot be ascribed to contamination unless a selective contamination exists.
- As detailed before, all over the world MMTV has been examined in 51 BC populations and found present in 82% of them. The percentage of positivity remains between 18% and 28%, with the only exception of North Africa and Australia, where it reaches 40%.

### 14.2. HERVs

One of the preferred claims against the existence of HMTV is that the presence in human tissues of HERVs similar to MMTV can induce false positive results.

Some of the most relevant papers on HMTV solved the problem by a careful comparative analysis of the viral sequences used in their experiments with HERVs sequences [125,126]. The methodology followed in the paper on the discovery of HMTV in ancient human remnants was particularly accurate [228].

### 14.3. Other Issues

- MMTV tumors and human BC have different morphology.
  This statement has no scientific base. The reading of Thelma Dunn’s original article [82] and of a more recent paper by James Lawson et al. [178] can shed light on the similarities between human and murine tumors. However, it should be highlighted that both murine and human tumors have the same origin, in that they derive from the TDLU, the terminal duct lobular unite. In fact, the name “ductal carcinoma”, used for BC for a long time, was abandoned because it was misleading. It was substituted with “invasive carcinoma NST (no special type) or NOS (not otherwise specified)”. In fact, NST/NOS BC, even if it represents almost the 80% of invasive BCs, does not recognize specific histotypes [8].
- If HMLV has a similar life-cycle to MMTV, it would be expected that transmission via breast milk should occur. Epidemiological studies failed to demonstrate that breast-feeding was a risk factor for human breast cancer.
  As detailed in Section 21.1, biological reasons make it highly improbable that human milk can transmit the MMTV/HMTV/HBRV. This well fits with the reported epidemiological data.
- Traces of MMTV are detected in normal mouse breast tissues and epithelia.
  MMTV is well documented in human normal mammary tissues [149,193], as a result of the spreading of the virus in the host.
- Pregnancy has a well-known protective effect against the risk of developing breast cancer in humans: the opposite is true for MMTV caused murine hyperplasia.
  As detailed in Section 1.3.5, a late menarche, an early menopause, and pregnancies reduce BC risk in that the proliferative effect of estrogens on the mammary gland is reduced. It is a matter of pathogenesis and not of etiology; moreover, it does not apply to mice.
- Unlike all established human oncogenic viruses, chronic immuno-suppression does not predispose to human breast cancers.
  In the mouse, MMTV needs an efficient immune system, necessary to infect the host and for its distribution to the organs, and to mammary glands, in particular. Studies report that in immunosuppressed women the incidence of BCs either decreases or does not increase, in favor of the MMTV etiology [229,230,231,232,233].
- Human cells lack the receptor necessary for the viral entry of MMTV.
  As discussed in point 7.3, MMTV can infect human cells in absence of the TfR1 [185].
- The presence of the viral marker should be more frequent in cases than in normal controls in the same geographic setting, as measured by the virus present in human mammary epithelial cells, and antibodies to the virus being present/elevated.
  Point 6 illustrates the large number of studies worldwide, with results in line with these requests.
- Temporal relationship: exposure to the virus should precede disease outcome.
  This was demonstrated in 2017 [193].
- The association of the virus with breast cancer should be replicated by multiple investigators”.
  The association MMTV/human BC was shown in 42 different studies against nine with negative results, with 1320 positive cases out of 5015 (26%); see point 6.
- The geographical prevalence of the virus should be highest where the incidence of breast cancer is highest.
  This study is very difficult. The epidemiological data on cancer are not fully reliable due to differences among countries in terms of Cancer Registries, awareness campaigns, and early diagnosis (see point 20).
- There should be a dose–response relationship between exposure level and the incidence of breast cancer.
  Not applicable.
- Biological plausibility/coherence: viral causation should make sense in terms of mode of transmission, natural history/pathology, and oncogenic capacity (virus can infect human mammary epithelium, can transform human mammary epithelial cells, can induce malignancies in an animal model).
  The answer is positive for all these points, as illustrated in the different sections of this article.
- Negative data are consistent with the majority of papers on this topic, while only a few laboratories describe MMTV positive human breast-cancer specimens.
  This statement is not supported by the analysis carried out in this article and previously summarized.

Some of the cited papers gave special attention to the issue of contamination by adopting very accurate laboratory methodologies [125,126,193,228]. James Lawson and Wendy Glenn discussed this topic in detail in two recent papers [194,234], one of which is in this same issue of Viruses.

## 15. Breast Cancer Incidence in Immunosuppressed Patients

Quite interestingly, in several studies the incidence of BC is described as unchanged or even reduced in immunosuppressed women, such as HIV or transplant patients [229,230,231,232,233,235]. This fact is considered against a viral origin of BC, in that several viruses benefit from a situation of immunodeficiency.

As already discussed, MMTV has special relationships with the immune system of the mouse, which has to be working perfectly to allow the infection. 

Hence, the above epidemiological data are expected, following MMTV biology. Of course, these studies are observational and require a biological confirmation.

## 16. The Pisa Meetings

Until ten years ago some of the few scientists interested in demonstrating the existence of a human MMTV had never met. As a remedy, the author took the initiative to organize scientific meetings in Pisa.

### 16.1. 2012 (2–4 April): HMTV—Viruses and Breast Cancer

It was the first international meeting on MMTV in humans, under the auspices of the University of Pisa, the European Society of Pathology, and the Tuscan Regional Government.

The speakers were as follows: Gertrude Buehring, University of California, Berkley; Robert Callahan, NCI, NIH, Bethesda, MD; Jaquelin Dudley, University of Texas, Austin; Ezequiel Moises Fuentes Pananá, Hospital de Pediatriá, Mexico City; Walter Gunzburg, University of Veterinary Medicine, Vienna; Jacob Hochman, The Hebrew University of Jerusalem; James Lawson, University of New South Wales, Sydney; Andrew Mason, University of Alberta, Edmonton; Chiara Maria Mazzanti, University of Pisa; Stella Melana, Mount Sinai School of Medicine, New York; Mauro Pistello, University of Pisa; Beatriz Pogo, Mount Sinai School of Medicine, New York; Susan Ross, University of Pennsylvania, Philadelphia; Brian Salmons, Austrianova Singapore Pte Ltd., Singapore; Alexander Stewart, University of Ottawa; Harald zur Hausen, German Cancer Research Center, Heidelberg, and; the author.

The three conference days were full of constructive discussion and stimulating ideas and useful for planning future research. Harald zur Hausen closed the meeting with a synthesis of the presentations. The conclusion was that the quality and quantity of the data justified pursuing the hypothesis of the MMTV etiology of human breast cancer.

### 16.2. 2016 (22–23 September): HBRV—MMTV and Human Diseases

The following scientists took part in the event: Ori Braitbard, The Hebrew University of Jerusalem; Giulia Freer, University of Pisa; Eliyahu Golomb, The Hebrew University of Jerusalem; Nicole Grandi, University of Cagliari; Walter Gunzburg, University of Veterinary Medicine, Vienna; Jacob Hochman, The Hebrew University of Jerusalem; Stanislav Indik, University of Veterinary Medicine, Vienna; James Lawson, University of New South Wales, Sydney; Michael Lisanti, University of Salford, Manchester; Andrew Mason, University of Alberta, Edmonton; Chiara Maria Mazzanti, Fondazione Pisana per la Scienza; Stella Melana, Mount Sinai School of Medicine, New York; James Neil, University of Glasgow; Mauro Pistello, University of Pisa; Beatriz Pogo, Mount Sinai School of Medicine, New York; Brian Salmons, Austrianova Singapore Pte Ltd., Singapore; Enzo Tramontano, University of Cagliari, and; the author.

The existence of a human betaretrovirus involved in breast cancer and primary biliary cholangitis was considered in detail. The first data on the presence of HBRV in ancient humans were presented and discussed, giving more determination to carry on.

**Part IV: Human Betaretrovirus—HBRV**

During this first part of the century, two concepts became more solid and finally accepted: (a) there is a virus (MMTV/HMTV) well established in humans, strictly connected to breast carcinoma; (b) the same virus (or highly homologous) is strictly connected with the primary biliary cholangitis (PBC), a chronic, inflammatory, autoimmune disease.

As a consequence, the names used to date to indicate the virus were no longer appropriate, in that the virus was human and no longer identified only with BC. HBRV means Human BetaRetroVirus, and is well suited to the situation: a human virus, a betaretrovirus, a virus linked to different types of disease and potentially involved in other diseases.

## 17. The Human Species Has Its First Human Betaretrovirus

Two years ago, the author and his colleagues hypothesized that the finding of viral sequences in remnants of ancient individuals would have demonstrated the existence of a human MMTV/HMTV, in other words of the HBRV.

The strategy consisted in the analysis of 36 ancient skulls dated from the Copper Age to the 17th century, all from Italian regions, namely, Sardinia, Tuscany and Liguria. The dental calculus (tartar) was chosen as the material to be investigated. The choice was suggested by the previous demonstration of MMTV/HMTV in human saliva and salivary glands in that the calculus originates from saliva, which provides many constituents of the dental plaque, a bacterial biofilm covering the surface of teeth. Over time, plaque undergoes a process of calcification, giving origin to the calculus through gradual calcium phosphate mineralization. Interestingly, calculus is an excellent material for the analysis of the paleomicrobiome, in that it provides an excellent preservation of the nucleic acids of the microorganism remained entombed in it during the life. Moreover, the calculus is not easily colonized by environmental bacteria, does not have points of entry for exogenous bacteria and lacks nutrient sources that are able to attract bacteria and support their growth [236,237,238]. It is easily imaginable that in antiquity the teeth were covered by an abundant quantity of dental calculus.

Six of the 36 individuals examined revealed the presence of the *Env* sequences being investigated. Two of them were dated back to the Sardinian Copper Age, approximately 4500 years ago, while the other four were from the 6th–17th century [228].

Most importantly, an accurate bioinformatic analysis demonstrated that the amplified sequences do not derive from the amplification of HERV-K HML (human endogenous retrovirus-K human MMTV like). Moreover, the phylogenetic analysis of the amplified regions showed that all the amplicons grouped together with HMTV and MMTV C3H were phylogenetically well distinct from all the other exogenous and endogenous betaretroviruses.

HBRV was finally born.

## 18. Cross-Species Transmission

Pathogens can change their host, moving from one species to another. This phenomenon is called cross-species transmission (CST) or interspecies transmission or host jump or spillover. When CST causes a disease, it is called *zoonosis*, from the ancient Greek ζῷον (*zòon*, animal) and νόσος (*nósos*, disease). A CST can occur today or it could have occurred in ancient times. In the latter, the new host is permanent and the disease becomes typical of the new species.

Several modern diseases began in the past as a zoonosis, such as measles, small pox, influenza, diphtheria. As any pathogen can, retroviruses too can jump from animals to humans. An example is HIV, which became human at the beginning of the last century, moving from its original host, the chimpanzee [239].

Many animal pathogens became human pathogens about 10,000 years ago, at the beginning of civilization, in the area named the *fertile crescent*, which includes Western Asia, the Nile Valley and the Nile Delta, where and when agriculture arose. Humans began to cohabit with other species, some of which were then domesticated. Mice, having a guaranteed abundance of food and with a very high reproduction rate, became a very large population, beginning their long story of strict “friendship” with man. In this so promiscuous habitat, animal pathogens began to infect humans to definitively settle within them. The rise of agriculture is considered a key moment for CST [240].

In the past millennia there was a rise in the frequency of house mice with the intensification of urbanization. Cats became domestic to control rodents and possibly participated in giving rise to the origin of zoonoses [241].

## 19. HBRV as the Product of a Cross-Species Transmission

The finding of HBRV in our ancestors from thousands of years ago is highly indicative that a mouse-human cross-species transmission occurred in the fertile crescent in prehistoric times. This process led to the humanization of MMTV, which finally became HBRV.

## 20. The Possibility of a Contemporary Zoonosis

Bearing in mind the murine model, the following hypothesis comes spontaneously: mice host the MMTV, mice are everywhere, ergo mice transmit viruses to humans.

In 2000, Thomas Stewart et al., [242] published a paper with the intriguing title “Breast cancer incidence highest in the range of one species of house mouse, Mus domesticus”. The key points of the study are as follows:

(a) The two most numerous subspecies of *Mus musculus*, *Mus m. musculus* and *Mus m. domesticus,* each have a different geographic distribution: *Mus m. musculus* lives in eastern Europe and in northern Asia, while *Mus M. domesticus* lives in western Europe, in north Africa, and in the near East. A third subspecies, *Mus m. castaneus*, lives in southeast Asia, from the eastern part of the Indian subcontinent to all southeastern Asia.
(b) The number of endogenous MMTV loci is higher in *Mus m. domesticus* than in *Mus m. musculus* and *Mus m. castaneus*; exogenous infectious particles can evolve from endogenous particles; as a consequence, *Mus m. domesticus* is more infectious for what concerns MMTV.
(c) The incidence of breast cancer is higher in western Europe in comparison to eastern Europe.
(d) The higher number of breast cancer patients in western Europe can be a consequence of a zoonosis, with a direct transmission to humans of MMTV from *Mus m. domesticus*.

The paper had a strong impact on the scientific community interested in the existence of a human MMTV and was an object of particular attention in the 2012 Pisa Meeting on Viruses and Breast Cancer. Very recently, Alexandre Stewart and Hsiao-Huei Chen revisited the MMTV zoonotic hypothesis, basically confirming their data of 2000 [243].

The hypothesis that mice can transmit MMTV to humans makes sense, not least for the billions of mice living on the earth. There are, however, a few points that deserve attention:

(a) Knowledge about the presence of MMTV in wild mice is quite limited, with studies usually conducted on exiguous numbers of animals. However, exogenous MMTV has been found in all the three Mus subspecies mentioned, *M. musculus*, *M. domesticus*, *M. castaneus* [224,242]. In particular, percentages of positivity are quite similar in *m. domesticus* (50%) and *M. musculus* (43%) [242].
(b) According to Murray B. Gardner, “the prevalence of type B virus (mammary tumor virus) and of spontaneous breast tumors was uniformly low in all the population of wild mice” and “in wild mice MMTV is transmitted by milk and not genetically, exists in low prevalence, causes a few breast tumors, and plays an insignificant role in the overall biology or causes of death” [244].
(c) In recent times, several papers indicate that BC epidemiological data are changing [245,246,247,248,249]. There is an increase in the BC incidence, globally and, in particular, in eastern countries that reach levels similar to those of western countries. These changes are attributable, at least in part, to the development of cancer registries and to better screening programs, but also to the genetics of the populations, to reproductive risk factors such as earlier age at menarche, delayed age at first birth, reduced breastfeeding, and declining fertility rates.
(d) Moreover, a classic assumption of BC epidemiology is now in question: the change in BC incidence in individuals moving from one country to another with different frequency of the disease is currently under debate [250,251].
(e) Finally, the paleoantrophological data cannot be ignored [228].

In any case, the two situations are not necessarily mutually exclusive and could coexist.

## 21. Inter-Human Transmission of HBRV

Ascertaining the human nature of HBRV: how is the infection transmitted?

### 21.1. Milk (Breast Feeding) Cannot Be the Answer

As seen in the previous parts of this article, scientists for a long time, and in vain, have tried to demonstrate the presence in human milk of MMTV/HMTV in a quantity sufficient to infect the newborn. This result is in line with what is known about the biological basis of infection in mice:

- To infect the newborn mouse, a large quantity of virus is required. Because at any lactation, most of the particles are denatured in the stomach [47,48,252], the critical quantity of virus necessary to reach the ileum and infect the host entering the Peyer’s patches is reached with incoming lactations.
- To obtain a high incidence of mammary tumors by nursing, it is necessary to nurse for an appreciable period of time, despite that during the first few days, milk is not at all deficient in virus, containing about 10^12^ virions/mL [252].
- Human milk has been shown to exert a destructive effect on MMTV particle [114,253].
- B particles are very difficult to find in human milk [253].

However, the presence in human milk of traces of MMTV in terms of viral particles, viral sequences, and reverse transcriptase [106,109,254,255], is evidence of the existence of the HBRV in our species.

### 21.2. Retroviruses (MMTV Included) Can Be Transmitted by Saliva in Animals

In the mouse and in other animals, MMTV as well as exogenous retroviruses, in general, are present in salivary glands and saliva, and can be transmitted horizontally [96,256,257].

### 21.3. MMTV Can Infect Adult Mice through the Nose

Waldeyer’s ring is, in humans, a major defensive system against microbiological agents (ingested or inhaled) situated at the main entry of the body. It is made by several tonsils (pharyngeal/adenoids, tubal, palatine, lingual), lymphoid immunocompetent organs. The tonsils’ structure and function are quite similar to those of Peyer’s plaques, discussed previously. M cells uptake antigens and stimulate B and T cells [258]. NALT (naso-pharingeal associated lymphatic tissue) is the equivalent of the ring in the mouse.

Considering that a lymphoid tissue is indispensable for MMTV to infect the newborn mouse and to spread into the host, in 1997, Dominique Velin et al. [85] wondered if a different lymphatic structure could serve the same function, choosing NALT. Hence, they were successful in infecting adult mice by nasal administration of MMTV-infected milk.

### 21.4. HBRV Is Present in Human Saliva

Human saliva and salivary glands contain HBRV. Fluorescent PCR, RT-PCR, FISH, immunohistochemistry, and the whole transcriptome analysis were used [190].

The saliva of newborns is virus-free, while viral sequences were present in 27% of children, in 11% of healthy adults, and in 57% of breast cancer patients. Eight percent of salivary glands was also positive. RNA-seq analysis performed on the saliva of a breast cancer patient demonstrated a high expression of MMTV RNA in comparison to negative controls.

It is noteworthy that: (a) the absence of viral sequences in the saliva of newborns eliminates the possibility of a trans-placental transmission and strongly supports an inter-human transmission; (b) the saliva and the salivary glands of adults have a very similar percentage of positivity (11% and 8%, respectively); (c) the fact that the percentage of positive cases in pediatric ages is almost three times that of adults does not surprise, considering that children are much more prone than adults to infections; (d) the very high positivity in breast-cancer patients is a highly significant link between the virus and breast carcinoma.

## 22. HBRV Transmission by Saliva

All the data here summarized make conceivable the possibility that HBRV can be spread into a population by the saliva of an infected person. The virus can easily reach the Waldeyer’s ring structures. The oro-pharingeal lymphatic structure can be an entry-side for HBRV, as seen before.

### 22.1. Saliva Contains a Little Universe

Saliva and its droplets contain viruses, bacteria, epithelial cells, immunocompetent cells, molecules of different type, and ions [259].

### 22.2. Saliva Spreading

Saliva transmits its content by contact or by air (aerosol, droplets) [260].

### 22.3. Saliva Transmits Even Prions

Saliva transmits prions and the diseases they cause. This happens in chronic wasting disease (CWD) in cervids, a very interesting natural model of neurodegenerative disease quite similar to Alzheimers. Moreover, despite the very low salivary concentration of prions, a single exposure can initiate infection [261].

### 22.4. What Is in Saliva That Can Diffuse HBRV

This is a difficult question to answer. However, the betaretrovirus HBRV is a “slow” virus with a special link with the immunocompetent cells, which it uses as a “taxi”. So, a situation different from that of the more common viruses can be expected. Are there complete viral particles in a small but sufficient quantity? Do the lymphocytes contain viral nuclei acids? Do the epithelial cells contain viral nuclei acids? Are there free viral nucleic acids? Indeed, all these questions are waiting for definitive answers.

## 23. Breast Cancer in the Same Family: A Transmissible Agent?

The appearance of breast cancer in genetically unrelated individuals of the same family has been described several times. Most cases deal with wife and husband, but there is also a case with father, mother, and daughter. These reports [262,263,264,265,266] surely stimulate the hypothesis of the presence of a transmissible agent. However, they do not contain information about the genetics of the family, making it impossible to rule out the possibility of a genetic transmission of BC. The fact that the contribution of germ line BRCA1/2 mutations to male BC risk is significant has to be mentioned [267]. In any case, these families deserve to be the object of specific genetics and molecular studies to avoid remaining the subject of anecdotal reports.

## 24. The Abundance of BC Histotypes and Biological Types can Find a Reason in a Viral Etiology

The term “ductal” carcinoma has now been disproven and thus mothballed. In fact, the site of origin of BC is the TDLU and not the ducts, for reasons explained before.

Up to 80% of invasive carcinoma are today called “no special type” (NST) or “not otherwise specified” (NOS); invasive lobular carcinoma represents about 10%; the others, about twenty histotypes, are about the remaining 10% under the definition of “special types” invasive carcinoma [8].

Actually, NSTs, in turn, have a large number of architectures, difficult to catalog somehow, made of a variable quantity of acini, solid groups of cells, and stroma, often in large quantities. Moreover, the analysis of hormone receptors and of HER2 expression allows a further examination with prognostic/therapeutics purpose.

The question “why this richness of architectures?” is legitimate and might be explained by a viral etiology of the disease, for a couple of possible reasons by analogy to the situation in mice, which have been discussed before:

(a) The morphology of murine mammary tumors is determined by the strain of virus and not from the genetics of the host.
(b) MMTV exerts its oncogenic action in mice primarily by a random process of insertional mutagenesis, with certain trigger integrations leading to the activation of many pathogenetic molecular pathways.

In respect to the latter point, it is noteworthy that “MMTV displays the most random dispersion of integration sites among retroviruses determined so far” [268].”

Hence, two explanations are possible and deserve to be investigated:

(1) Different strains of HRBV exist, and are able to activate oncogenesis in different ways.
(2) HRBV adopts the mechanism of insertional mutagenesis, activating a various and wide number of pathways, with different morphological and biological consequences.

Preliminary data from the Pisa laboratory indicate the presence of HBRV in almost all the histotypes.

## 25. Oncogenesis of HMTV

On the basis of what is known about MMTV, it is possible that HBRV carries out its oncogenetic mechanism through a process of insertional mutagenesis. MMTV INT and CIS have been of great relevance in the studies on human BC, with the identification of genes and pathways related to BC and to general carcinogenesis [73,268,269,270,271,272,273,274,275,276,277,278,279,280,281,282,283]. Unfortunately, precise, complete pathways have not yet been defined. The introduction and development of the MMTV transgenic mouse models hopefully will be of help in the future [284,285].

In the meantime, mechanisms other than insertional mutagenesis could play a role, at least in theory, as discussed by Salmons and Gunzburg [286,287]:

### 25.1. Proteins with Characteristics of Oncogene

#### 25.1.1. The Env Protein

The surface unit (SU) of the Env protein hosts an ITAM sequence, which has tyrosine residues phosphorylated by Src kinases. Src recruits Syk kinases, able to phosphorylate substrates involved in cell proliferation and differentiation. ITAMs are encoded also by viruses as EBV and BLV. The ITAM associated to Env is able to initiate malignant transformation of mammary epithelial cells [76,77]. Interestingly, the Env of another Betaretrovirus, JSRV, behaves as an oncogene in ovine pulmonary adenocarcinoma (OPA) [288].

#### 25.1.2. The Sag, Superantigen

MMTV has an open reading frame (ORF) in the 3′ LTR, encoding also for Sag, indispensable for the infection of lymphocytes and the diffusion of infection. *Sag* sequences isolated from human BC were shown to be able to perform functions similar to those in the mouse [130]. Then, it was observed that *Sag* may act as an oncogene, stimulating the proliferation of epithelial cells and promoting tumorigenicity of hyperplastic mammary epithelial cells [289].

#### 25.1.3. The Naf, Negative Acting Factor

*Naf* is hosted by the same ORF. Actually, *sag* and *naf* share parts of their coding sequences. Naf is a transcriptional repressor of retroviral genome and is able to induce differential expression of several proteins and to reduce epithelial cell growth. These characteristics suggest a possible role in oncogenesis [67,290,291].

#### 25.1.4. The REM (Regulator of Expression/Export of MMTV mRNA)

Rem is a Rev-like protein (**r**egulator of **e**xpression of **v**irion proteins) present in the nucleus and involved in the export of unspliced, or incompletely spliced, mRNA. Rem is an alternative splice variant of the *env* mRNA localized to nucleoli. p14, a 14-kDa phosphoprotein, is the Rem-derived signal peptide. Of particular interest are two phosphorylation sites at Serines 18 and 65, phosphorylated by two different kinases of the PKC and CK2 types, respectively. Interestingly, p14 can function as either an oncogene, when phosphorylated by CK2, or as a tumor suppressor, when phosphorylated by PKC [191,292].

### 25.2. Cell Fusion

Viruses can spread between host cells by creating physical doors of communication through fusion of their cell membranes. Tetraploidy is a consequence of cell fusion, which can give origin to cancer-associated aneuploidy. A mechanism of control is a p53 and pRb dependent growth arrest of a tetraploid cell. Unfortunately, human oncogenic viruses, included retroviruses, can inhibit p53 and pRb, giving origin to a pathogenic chromosomal instability. Interestingly, tetraploid cells are more frequent in virus-positive than in virus-negative premalignant lesions [293]. Cell fusion has been known in cells infected by MMTV, for a long time [294,295].

### 25.3. Activation of a Second Oncogenic Virus

MMTV could activate another virus, maybe already known in human oncogenesis and in some way associated to BC, such as EBV and HPV. An example is the HIV-1 Tat that activates KSHV (Kaposi’s sarcoma-associated herpesvirus) by regulating PI3K/PTEN/AKT/GSK-3b pathway [296].

### 25.4. HERVs

There may be an interaction between HBRV and HERVs (e.g., a reciprocal activation) or even the production of pseudotyped HBRV and HERVs virions or retrotranspositions that may also play a role in the causation of breast cancer [216,297].

## 26. How Many HBRV?

HBRV is linked to BC. At the same time, it is linked to PBC. Moreover, BC has a myriad of histotypes, possibly related to the virus.

In the mouse, several types of MMTV exist, able to give origin to tumors different in terms of morphology and biology.

The possibility that different strains of HBRV exist has to be taken into consideration.

**Part V**

## 27. Keynotes

1. A few million new cases of breast cancer are diagnosed worldwide every year.
2. No cause is known for breast cancer. Estrogens are essential pathogenetic factors, but their etiological role has never been proven. No sound etiological hypothesis alternative to the viral one has ever been advanced.
3. The betaretrovirus MMTV, the mammary tumor virus, is a potent oncogenic agent that in the mouse causes mammary tumors, and also lymphomas.
4. The experimental model of MMTV-induced murine mammary tumors had a significant role in unveiling central aspects of the biology of cancer, in general, and of human breast cancer, in particular.
5. Murine mammary tumors and human breast carcinomas have unquestionable biological and morphological similarities.
6. MMTV needs an efficient immune system to infect the host and to spread to its organs.
7. A vast and solid amount of data collected worldwide indicates the existence of MMTV in humans, therefore named HMTV, human mammary tumor virus. At the same time, data strongly indicate the involvement of HMTV in human breast cancer and in PBC, primary biliary cholangitis.
8. HMTV is detectable in normal breasts years before the appearance of cancer; moreover, it is present in preinvasive breast lesions, with 80% positivity in ductal carcinoma in situ (DCIS), whereas it is about 40% in invasive tumors.
9. A paleoanthropological study has demonstrated that HMTV is a human virus living with humans over thousands of years.
10. This “new” virus, actually the first human Betaretrovirus, is named simply HBRV, human betaretrovirus, in that it is not any more related only to the mouse or to mammary tumors, but also to PBC, an inflammatory liver disease.
11. HBRV could be spread by saliva and infect through the Waldeyer’s ring lymphatic structures.
12. Human cancers with a viral etiology are numerous.

## 28. For the Future

The evidence for a causal role of HMTV/HBRV in human breast cancer has continued to increase over the last 25 years due to the advances in technology and methodology, on which we count for its conclusive demonstration.

The hope is that a century of passionate and enthusiastic research can motivate the scientific community to invest in this goal.

## Figures and Tables

**Table 1:** Worldwide presence of HMTV in human breast carcinoma.

Continent	Country	Year	Cancers			Tissue Preservation		1st Author	Ref.
			Total	No. +	% +				
**North America**	**United States**	1995	314	121	**38.5**	frozen		Wang	[125]
			151	60	**40**	FFPE			
		2000	73	27	**37**	frozen		Etkind	[135]
		2001	106	32	**30**	FFPE		Melana	[136]
		2001	65	27	**41.5**	frozen		Wang	[129]
		2003	484	178	**37**	FFPE or frozen		Wang	[137]
			29	18	**62**		gestational		
		2004	12	6	**50**	FFPE	associated lymphoma	Etkind	[138]
	Total		1234	469	**38**				
	**Mexico**	2007	119	5	**4.2**	frozen		Zapata-Benavides	[139]
		2013	86	0	**0**	frozen		Morales-Sánchez	[140]
		2014	458	57	**12.4**	frozen		Cedro-Tanda	[141]
	Total		663	62	**9**				
**Total**			**1897**	**531**	**28**				
**South America**	**Argentina**	2002	74	23	**31**	fresh, FFPE		Melana	[142]
	**Brasil**	2020	217	41	**19**	fresh		de Sousa Pereira	[143]
**Total**			**291**	**64**	**22**				
**Europe**	**Austria**	2003	50	0	**0**	frozen		Witt	[144]
	**Croatia**	2021	70	5	**7**	FFPE		Gupta	[145]
	**Italy**	1999	69	26	**38**	FFPE		Pogo	[146]
		2002	17	0	**0**	frozen micro-dissection		Zangen	[147]
		2006	45	15	**33**	frozen		Zammarchi	[148]
			Pure and enriched population of epithelial cells by laser microdissection. Fluorescent-nested PCR.						
		2011	20	7	**35**	FFPE microdissection		Mazzanti	[149]
		2019	56	17	**30**	FFPE microdissection		Naccarato	[150]
	Total		207	65	**31**				
	**Sweden**	2007	18	0	**0**	frozen		Bindra	[151]
	**UK**	2004	44	0	**0**	frozen		Mant	[152]
**Total**			**389**	**70**	**18**				
**Africa**	**Egypt**	2013	100	36	**36**	FFPE		Hafez	[153]
		2021	50	38	**76**	frozen		Loutfy	[154]
		2021	40	28	**70**	frozen		Metwally	[155]
			40	3	**7.5**	FFPE			
	Total		230	105	**46**				
	**Morocco**	2014	42	24	**57**	FFPE		Slaoui	[156]
	**Tunisia**	2004	38	28	**74**	FFPE		Levine	[157]
		2008	122	17	**14**	frozen		Hachana	[158]
	Total		160	45	**28**				
**Total**			**432**	**174**	**40**				
**Asia**	**China**	2006	131	22	**17**			Luo	[159]
		2021	119	21	**18**	FFPE		Wang	[160]
			84 n		**22.62**				
			35 s		**5.71**				
			n: North of China; s: South of China						
	Total		250	43	**17**				
	**Iran**	2012	50	0	**0**	FFPE		Motamedifar	[161]
		2013	40	0	**0**	FFPE		Tabriz	[162]
		2014	65	0	**0**	FFPE		Ahangar	[163]
		2015	100	12	**12**	FFPE		Reza	[164]
		2017	59	19	**32**	FFPE		Shariatpanahi	[165]
		Provinces of Fars, Tehran, East Azerbaijan, Kerman, and Isfahan, respectively, in the South-West, North-Center, North-West, South East and Center of the Country.							
	Total		314	31	**10**				
	**Japan**	2008	46	0	**0**	frozen		Fukuoka	[166]
	**Jordan**	2020	100	11	**11**	FFPE microdissection		Al Hamad	[167]
	**Pakistan**	2014	80	21	**26**	FFPE		Naushad	[168]
		2017	50	19	**38**	FFPE		Naushad	[169]
		2017	250	83	**29**	FFPE		Naushad	[170]
		2021	105	69	**66**			Khalid	[171]
	Total		485	192	**40**				
	**Republic of Korea**	2019	128	12	**9**	frozen		Seo	[172]
	**Saudi Arabia**	2018	103	9	**9**	FFPE		Al Dossary	[173]
			Saudi: 4+/67 patients, 6%; Non-Saudi: 5+/36, 14%						
	**Vietnam**	2003	120	1	**0.8**	FFPE		Ford	[174]
**Total**			**1546**	**299**	**19**				
**Australia**		2003	45	19	**42**	FFPE		Ford	[174]
			Caucasian-Australian						
		2004	128	50	**39**	FFPE		Faedo	[175]
		2004	136	43	**32**	FFPE		Ford	[176]
		2004	42	20	**48**	FFPE		Lawson	[177]
		2006	59	22	**37**	FFPE		Lawson	[178]
		2008	50	28	**56**	FFPE		Mok	[179]
**Total**			**460**	**182**	**40**				
**Total General**			**5015**	**1320**	**26%**				
